# Nitrogen regulation of protein–protein interactions and transcript levels of GlnK PII regulator and AmtB ammonium transporter homologs in Archaea

**DOI:** 10.1002/mbo3.120

**Published:** 2013-08-28

**Authors:** Laia Pedro-Roig, Christian Lange, María José Bonete, Jörg Soppa, Julie Maupin-Furlow

**Affiliations:** 1 Departamento de Agroquímica y Bioquímica, Facultad de Ciencias, Universidad de Alicantecarretera de San Vicente s/n, 03080, Alicante, Spain; 2 Institute for Molecular Biosciences, Johann Wolfgang Goethe UniversityMax-von-Laue-Strasse 9, 60438, Frankfurt a.M, Germany; 3 Genetics Institute and Department of Microbiology and Cell Science, University of FloridaGainesville, Florida, 32611

**Keywords:** Archaea, *Haloferax*, halophilic, membrane, metabolism

## Abstract

Gene homologs of GlnK PII regulators and AmtB-type ammonium transporters are often paired on prokaryotic genomes, suggesting these proteins share an ancient functional relationship. Here, we demonstrate for the first time in Archaea that GlnK associates with AmtB in membrane fractions after ammonium shock, thus, providing a further insight into GlnK-AmtB as an ancient nitrogen sensor pair. For this work, *Haloferax mediterranei* was advanced for study through the generation of a *pyrE2*-based counterselection system that was used for targeted gene deletion and expression of Flag-tagged proteins from their native promoters. AmtB1-Flag was detected in membrane fractions of cells grown on nitrate and was found to coimmunoprecipitate with GlnK after ammonium shock. Thus, in analogy to bacteria, the archaeal GlnK PII may block the AmtB1 ammonium transporter under nitrogen-rich conditions. In addition to this regulated protein–protein interaction, the archaeal *amtB-glnK* gene pairs were found to be highly regulated by nitrogen availability with transcript levels high under conditions of nitrogen limitation and low during nitrogen excess. While transcript levels of *glnK-amtB* are similarly regulated by nitrogen availability in bacteria, transcriptional regulators of the bacterial *glnK* promoter including activation by the two-component signal transduction proteins NtrC (GlnG, NRI) and NtrB (GlnL, NRII) and sigma factor σ^N^ (σ^54^) are not conserved in archaea suggesting a novel mechanism of transcriptional control.

## Introduction

Members of the PII signal transduction protein family are found in oxygenic phototrophic eukaryotes, nearly all taxonomic groups of bacteria, and select archaea (primarily methanogens and halophiles) (Sant'Anna et al. 2009; Pedro-Roig et al. 2011). PII proteins regulate the assimilation of nitrogen compounds by interacting noncovalently with target proteins (Chellamuthu et al. 2013; Huergo et al. 2013). Regulation of PII targeting is typically in response to the availability of energy, carbon, and nitrogen and appears mediated by conformational changes in PII induced by metabolite (ATP, 2-oxogluatarate, and ADP) binding (Radchenko and Merrick 2011). In proteobacteria, PII proteins are also regulated by UMP modification (uridylylation) as catalyzed by the bifunctional GlnD (uridylyltransferase/uridylyl-removing) enzyme in response to nitrogen availability (Mangum et al. 1973; Adler et al. 1975; Jiang et al. 1998; Zhang et al. 2010). Recent studies have determined that the PII homologues in the halophilic archaeon *Haloferax mediterranei* are also subject to this kind of modification (Pedro-Roig et al. 2013a).

Multiple groups of PII proteins are now identified including GlnB/GlnK- and NifI-type regulators (Sant'Anna et al. 2009; Huergo et al. 2013). Of these groups, GlnK and GlnB are closely related and form homotrimers that associate with and regulate various enzymes, transcriptional regulators, and transporters (Chellamuthu et al. 2013). In particular, GlnK proteins often associate with and inhibit high-affinity ammonium transporters (e.g., AmtB) when the levels of ammonium are high (Coutts et al. 2002; Maier et al. 2011). Likewise, GlnB can interact with and stimulate the activity of dinitrogenase reductase ADP-ribosyltransferase (DraT), which mediates the ADP-ribosylation and inactivation of NifH (Moure et al. 2013). GlnB also associates with and regulates activity of the adenyltransferase (ATase) that covalently modifies and inactivates glutamine synthetase (Mangum et al. 1973). In addition, GlnB forms a complex with the bifunctional histidine kinase/phosphatase NRII (NtrB) that controls the phosphorylation status of its cognate response regulator NRI (NtrC) and, thus, regulates expression of the Ntr regulon important for the assimilation of ammonium and the assimilation of nitrogen compounds (Weiss et al. 2002). Of the NifI-type proteins, those of the methanogenic archaeon *Methanococcus maripaludis* are best characterized, where a model for ammonium-induced inhibition of nitrogenase activity through physical association of a NifI1-NifI2 complex with nitrogenase (NifDK) is demonstrated (Dodsworth and Leigh 2007).

The co-occurrence of GlnK PII and AmtB ammonium transporter gene homologs is common among prokaryotes including bacteria and archaea (Thomas et al. 2000; Sant'Anna et al. 2009; Huergo et al. 2013). These widespread genomic linkages suggest that the functional association of GlnK and AmtB first observed for *Escherichia coli* and *Azotobacter vinelandii* (Coutts et al. 2002) is common among prokaryotes from both domains of life. Consistent with this finding, association of GlnK and AmtB has also been experimentally shown for the bacteria *Bacillus subtilis* (Detsch and Stülke 2003), *Corynebacterium glutamicum* (Strösser et al. 2004), *Azospirillum brasiliense* (Huergo et al. 2006), *Rhodobacter capsulatus* (Tremblay et al. 2007), *Rhodospirillum rubrum* (Zhang et al. 2006), and *Herbaspirillum seropedicae* (Huergo et al. 2010). Nevertheless, no experimental proof of such an interaction exists for Archaea.

The GlnK and AmtB homotrimers have distinct structural features that provide insight into their associations. Typically, each subunit of GlnK is around 120 amino acids and is composed of two α-helices, six β-strands, and three loops (B-loop, C-loop, and T-loop) (Xu et al. 1998). AmtB transporters have 11 or 12 transmembrane helices (TMH) per subunit that are arranged in the homotrimer so that a pore for ammonium entry into the cell is created within each subunit (Khademi et al. 2004; Zheng et al. 2004). Crystal structures of *E. coli* AmtB:GlnK complex at atomic resolution provide insight into how binding of GlnK to AmtB regulates the ammonia channel thereby controlling ammonium influx in response to the intracellular nitrogen status (Conroy et al. 2007; Gruswitz et al. 2007). The T-loops of each subunit within the GlnK homotrimer protrude and are inserted into the three ammonia channels, so that they physically block the entrance of the nitrogenous substrate (Conroy et al. 2007; Gruswitz et al. 2007). This AmtB-GlnK interaction is suggested to be an ancestral form of ammonium sensing, as the *amtB*-*glnK* gene pair is one of the most highly conserved features of nitrogen control (Javelle and Merrick 2005).

Of the extreme halophilic archaea or haloarchaea, nitrogen metabolism is best understood in *Hfx. mediterranei* (Bonete et al. 2008), as this species can assimilate inorganic (ammonium, nitrite, and nitrate) and organic nitrogen compounds into cell carbon and is a denitrifier, able to use nitrate as final electron acceptor (Martínez-Espinosa et al. 2006). These features together with applications in brine decontamination (Najera-Fernandez et al. 2012) make *Hfx. mediterranei* an ideal model organism for the study of nitrogen metabolism and regulation in the haloarchaea. Our recent work demonstrated that the glutamine synthetase of *Hfx. mediterranei* is activated in vitro by association with PII GlnK-type regulators (Pedro-Roig et al. 2013b). Here, we report on yet another role of PII proteins that is likely to be conserved in haloarchaea, which is GlnK regulation of ammonium incorporation into the cell through AmtB-type membrane transporters. A system for the generation of *Hfx. mediterranei* knockout and knockin mutants was also developed, which proved highly efficient and useful for construction of the mutant strains of this study. Analysis of *glnK1* and *glnK2* gene transcripts of *Hfx. mediterranei* is also described.

## Experimental Procedures

### Strains and growth conditions

*Hfx. mediterranei* strains (Table 1) were routinely grown at 42°C with aeration at 225 rpm in orbital shakers placed in an enlightened room. Cultures were grown in a 25% (w/v) mixture of inorganic salts (25% SW) (Rodriguez-Valera et al. 1980) supplemented with 0.5% (w/v) yeast extract for complex medium and 0.5% (w/v) glucose and nitrogen source (5 or 75 mmol/L KNO_3_, NH_4_Cl or none) for minimal media. For exchange of nitrogen source, cells were harvested and washed twice with 25% SW at room temperature prior to inoculation into fresh medium. For mutant construction, a defined medium with 0.25% (w/v) casamino acids and 10 mmol/L NH_4_Cl was used. Medium was supplemented with 50 μg mL^−1^ uracil, 750 μg mL^−1^ 5-fluoroorotic acid (5-FOA), and 0.4 μg mL^−1^ novobiocin as needed.

**Table 1:** Strains and plasmids used in this study

Strain or plasmid	Description	Purpose	Source
*Haloferax mediterranei* strains			
R4	Wild type (ATCC 33500)	Physiology studies	DSMZ Bacteria Collection
HM26	R4 *ΔpyrE2*	Mutant construction	This work
HM26-K1	HM26 *ΔglnK1*	GlnK function studies	
HM26-K2	HM26 *ΔglnK2*		
HM26-K1K2	HM26 *ΔglnK1 ΔglnK2*		
HM26-F1	HM26 *flag:amtB1*	Amt-GlnK interaction studies	
HM26-F2	HM26 *flag:amtB2*		
HM26-F3	HM26 *amtB1:flag*		
HM26-F4	HM26 *amtB2:flag*		
Plasmids			
pTA131	3.6 kb, integration vector *Hfx. volcanii pyrE2* gene	Cloning	(Allers et al. 2004)
pMH101	3.6 kb, pTA131 with additional recognition sites for *Sna*BI, *Swa*I, and *Pme*I	Cloning	(Hammelmann and Soppa 2008)
pMH101N	5.9 kb, pMH101+ *gyrB*	Deletion mutants construction	This work
pMH101N-*ΔpyrE2*	6.9 kb, *pyrE2* deletion cassette		
pMH101N-*ΔglnK1*	6.9 kb, *glnK1* deletion cassette		
pMH101N-*ΔglnK2*	6.9 kb, *glnK2* deletion cassette		
pTA131-L1	4.6 kb, 1 kb around *amtB1* start codon	*flag:amtB* knockin mutants construction	This work
pTA131-L2	4.6 kb, 1 kb around *amtB2* start codon		
pTA131-L3	4.7 kb, 1 kb around *amtB1* stop codon		
pTA131-L4	4.7 kb, 1 kb around *amtB2* stop codon		
pTA131-L5	4.6 kb, 1 kb around *amtB1* start codon with an inserted Flag-tag		
pTA131-L6	4.7 kb, 1 kb around *amtB2* start codon with an inserted Flag-tag		
pTA131-L7	4.7 kb, 1 kb around *amtB1* stop codon with an inserted Flag-tag		
pTA131-L8	4.7 kb, 1 kb around *amtB2* stop codon with an inserted Flag-tag		

### Transformation protocol

*Hfx. mediterranei* was transformed using a method developed for *Hfx. volcanii* (Cline et al. 1989) with the following modifications: cells were grown to an OD_600_ of 0.2–0.5 units prior to harvest, 1.5 mol/L NaCl was added to the spheroplasting solution, 75 mmol/L EDTA (ethylenediaminetetraacetic acid) was used to form spheroplasts, and polyethylene glycol 600 was at 25% (w/v) final concentration.

### Mutant construction

*Hfx. mediterranei* mutant strains (Table 1) were constructed using a *pyrE2*-based ‘pop-in/pop-out’ strategy similar to previously reported for *Hfx. volcanii* (Bitan-Banin et al. 2003; Allers et al. 2004). Plasmids and primers used for targeted mutagenesis (including gene deletion and in-frame insertion of affinity tags onto the genome) are listed in Table 1 and Table S1, respectively. The method of restriction selection cloning (Russek et al. 1993) was used to fuse gene deletion cassettes (500-bp fragments of genomic DNA flanking the 5′ and 3′-ends of the gene to be deleted) into the *SnaB*I (pMH101N-*ΔpyrE2* and pMH101N-*ΔglnK2*) and *Pme*I (pMH101N-*ΔglnK1*) restriction sites of plasmid pMH101N. Plasmid pMH101N was constructed as a vector for *Hfx. mediterranei* gene deletions by cloning the Hfx. lucentense gyrB* novobiocin resistance gene into BamHI and XbaI sites of plasmid pMH101, which lacks an origin of replication for haloarchaea (Hammelmann and Soppa 2008). Plasmid pTA131, which carries a *pyrE2* gene (Allers et al. 2004), served as the vector for generation of the plasmids used for in-frame Flag-tag insertion of the *amtB1/2* target genes by the pop-in/pop-out method as follows. DNA fragments (1 kb) with sequence flanking the target gene translational start or stop codons were amplified by polymerase chain reaction (PCR) from wild-type (R4) genomic DNA using primers with restriction sites for *Hind*III or *Xho*I (forward primers) and *Bam*HI (reverse primers). The PCR products were ligated into the multiple cloning site of pTA131 (*Hind*III/*Xho*I to *Bam*HI) to generate plasmids pTA131-L1 to L4, which were used as template for inverse PCR to introduce a Flag-tag into the coding sequence of *amtB1/2* and generate plasmids pTA131-L5 to pTA131-L8. Primers for the inverse PCR contained the Flag-tag and a *Kpn*I linker.

HM26 (*ΔpyrE2*) was the parent strain used for all gene modifications and was derived from *Hfx. mediterranei* wild-type R4 (ATCC 33500) using plasmid pMH101N-*ΔpyrE2*. Plasmid pMH101N-*ΔpyrE2* was constructed by cloning the 5′ and 3′ flanking regions of *pyrE2* (∼500 bp each) into plasmid vector pMH101N. *Hfx. mediterranei* R4 cells were transformed with pMH101N-*ΔpyrE2*, and recombinants (pop-ins) were selected based on novobiocin resistance (see above section for details on medium composition). For selection of cells devoid of the *pyrE2* gene (pop-outs), recombinant cells (pop-ins) were subcultured in the presence of novobiocin for several generations and plated in defined medium supplemented with uracil and 5-FOA. All other pop-in/pop-out strains were generated by transformation of HM26 strain (*ΔpyrE2*) or its derivatives with pMH101-derived plasmids containing the *pyrE2* gene and selected for homologous recombination (pop-in) by plating in the absence of uracil. Strains undergoing a second step of homologous recombination (resulting in loss or pop-out of the plasmid) were selected by growth on medium with 5-FOA resulting in reversion to wild-type or targeted gene modification. 5-FOA resistant colonies were screened for targeted gene modifications by PCR and confirmed by Southern blot.

### Southern blot analysis

Genomic DNA (3 μg) was digested with appropriate restriction enzymes and separated by 1% (w/v) agarose gel electrophoresis in 1× TAE (Tris-acetate-EDTA) buffer. Separated DNA fragments were transferred overnight from the gel to a nylon membrane (Hybond-N+, GE Healthcare, Piscataway, NJ) using 20× SSC buffer (3 mol/L NaCl, 300 mmol/L sodium citrate pH 7.0) and cross-linked to the membrane with ultraviolet radiation. Prehybridization, hybridization, and chemiluminescent detection was performed as described in the DIG Application Manual for Filter Hybridization (Roche, Basel, Switzerland). Diagrams of the digestion strategy for the Southern blot analysis of the strains can be found in the Figures S1, S3, and S4.

### RNA methodologies

RNA was isolated using the RNeasy Mini Kit from QIAGEN (Hilden, Germany) following manufacturer's instructions and quantified by A_260_ using a NanoVue Plus spectrophotometer (GE Healthcare). Ethidium bromide staining of RNA samples separated by 0.8% (w/v) agarose gel electrophoresis in 1× TAE buffer was used to assess RNA integrity and served as a loading control for Northern blotting.

For analysis by Northern blotting, RNA samples were separated in a 1% (w/v) agarose gel containing formaldehyde. RiboRuler High Range RNA Ladder from Thermo (Waltham, MA) was used as size standard. RNA was transferred overnight to nylon membranes (Hybond-N+; GE Healthcare) by capillarity using 20× SSC buffer (3 mol/L NaCl, 300 mmol/L sodium citrate pH 7.0) and immobilized through covalent linkage using ultraviolet radiation. Cross-linked RNA fragments were probed against DIG-labeled PCR products (primers listed in Table S1). Prehybridization, hybridization, and chemiluminescent detection were performed according to the DIG Application Manual for Filter Hybridization (Roche). Separate membranes were used for each Northern blot.

For RT-PCR analyses (reverse transcription PCR), RNA samples were digested twice for 30 min at 37°C with the RNase-Free DNase Set from QIAGEN to avoid DNA contamination. RNA retrotranscription was performed using the iScript cDNA Synthesis Kit from Bio-Rad (Hecules, CA) and random hexamers. Regular PCR (Taq polymerase; Bioline, London, UK) was used to amplify the synthesized DNA with specific primers for *amtB1* and *amtB2* (primers listed in Table S1), which were designed in nonhomologous sequence regions from both genes (Fig. S9). A positive control with genomic DNA and negative controls without cDNA or with cDNA obtained from RT reactions lacking retrotranscriptase were also included. PCR fragments were visualized in 1.8% (w/v) agarose gels stained with ethidium bromide and compared to the 100 bp DNA Ladder from New England Biolabs (Ipswich, MA).

### Cell fractionation

Cells were harvested by centrifugation at 5500*g* for 15 min at 4°C (Avanti J-20 XP Centrifuge; Beckman Coulter, Brea, CA) and disrupted passing them twice at 2000 psi through a cooled French press (SLM AMINCO, Urbana, IL). Unbroken cells were removed by centrifugation at 5500*g* m for 15 min at 4°C and the supernatant was submitted to ultracentrifugation at 200,000*g* for 2 h at 4°C (L8-80M Ultracentrifuge, 70 Ti rotor; Beckman Coulter). The top clear fraction was used as cytoplasmic fraction and the pellet as membrane fraction. For immunoprecipitation 1 L cultures were harvested and cells were resuspended in 7 mL of 20 mmol/L Tris-HCl pH 8 buffer supplemented with 1 mol/L NaCl for disruption. Membrane pellets were washed twice with 20 mmol/L Tris-HCl pH 8 buffer supplemented with 1 mol/L NaCl and 2% (v/v) glycerol and resuspended in 1 mL of this same solution. For reversibility assays, 100 mL culture aliquots were used. Protein concentration was measured using the BCA assay (Pierce BCA Protein Assay Kit; Thermo Scientific).

### Immunoprecipitation

Triton X-100 was added at 2% (v/v) to resuspended membrane pellets and proteins were solubilized at 42°C for 2 h with gentle shaking. Nonsolubilized membranes were removed by ultracentrifugation (200,000*g*, 2 h, 4°C) and Anti-Flag M2 Affinity Gel (Sigma, St. Louis, MO) was added for overnight immunoprecipitation of AmtB1-Flag at room temperature. The agarose beads were washed with phosphate buffered saline supplemented with 0.02% (v/v) Triton X-100, and proteins were eluted by boiling in SDS-PAGE (sodiumdodecyl sulphate polyacrylamide gel electrophoresis) sample buffer without β-mercaptoethanol.

### Immunoblot

For GlnK detection, customized anti-GlnK polyclonal rabbit antibodies (Pedro-Roig et al. 2011) were used in combination with a peroxidase-labeled secondary antibody and the chemiluminescent substrate luminol (BM Chemiluminescence Western Blotting Kit from Roche). A commercial anti-Flag antibody (Monoclonal ANTI-FLAG M2-Alkaline Phosphatase Clone M2 from Sigma) and CDP-Star Chemiluminescent Substrate (Applied Biosystems, Foster City, CA) were used for Flag-AmtB identification.

### ICDH activity assay

Isocitrate dehydrogenase (ICDH) activity was used to determine the purity of the membrane fractions, as it is a cytoplasmic enzyme. Triplicates of assays performed in cytoplasmic and resuspended membrane fractions were compared. ICDH activity was assayed spectrophotometrically as described for *Hfx. volcanii* (Camacho et al. 1995).

## Results

### *Haloferax mediterranei amtB-glnK* gene homologs are linked

The halophilic archaeon *Hfx. mediterranei* possesses two GlnK PII gene homologs (*glnK1* and *glnK2*, 84% amino acid and DNA sequence identity) that are predicted to be encoded with homologs of Amt-type ammonium transporter genes (*amtB1* and *amtB2*, 73% DNA and 52% amino acid sequence identity), respectively (Pedro-Roig et al. 2011). This genetic linkage suggests that archaeal PII regulators interact with ammonium transporters, as has been demonstrated in a number of bacteria. Most haloarchaeal species (e.g., *Haloarcula marismortui* and *Haloquadratum walsbyi*) contain a single *amt-glnK* gene pair. By contrast, *Hfx. mediterranei* and *Hfx. volcanii* contain two *amt-glnK* pairs suggesting a gene duplication of *amt-glnK* which occurred rather recently in the genus *Haloferax* and may have resulted in the evolution of two *amt-glnK* gene clusters with specialized functions and/or differential modes of regulation.

### *Haloferax mediterranei glnK* and *amtB genes* are cotranscribed during conditions of ammonium limitation

**Figure 1:**
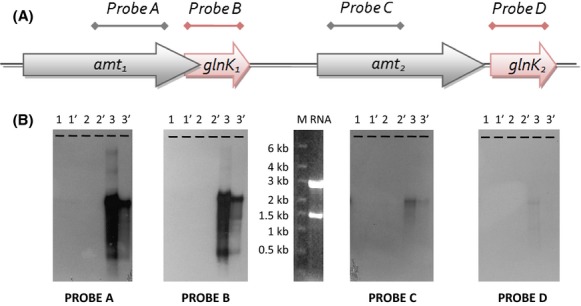
Northern blot analysis of the *amtB-glnK* genomic region of *Hfx. mediterranei*. (A) Four different DIG-labeled PCR products were used as probes. Primers (listed in Table S1) were amt1-mRNA-3F and amt1-mRNA-3R (probe A, 515 bp), glnK1-mRNA-1F and glnK1-mRNA-1R (probe B, 364 bp), amt2-mRNA-4F and amt2-mRNA-4R (probe C, 473), glnK2-mRNA-2F and glnK2-mRNA-2R (probe D, 337 bp). (B) 1 μg or 0.5 μg (‘samples) of RNA from *Hfx. mediterranei* wild-type cells grown with different nitrogen sources were probed with the four DIG-labeled PCR fragments. Lanes correspond to (1 and 1′) complex medium samples, (2 and 2′) 5 mmol/L NH_4_Cl samples, (3 and 3′) 5 mmol/L KNO_3_ samples, (M) *RiboRuler High Range RNA Ladder* from *Fermentas*, (RNA) RNA sample stained with ethidium bromide and visualized using ultraviolet light.

The two *amtB*-*glnK* gene pairs of *Hfx. mediterranei* were investigated at the transcriptional level. With this purpose, RNA samples were isolated from cells grown with different nitrogen sources (nitrate, ammonium, and yeast extract) and analyzed by Northern blotting using four probes, one for each gene (*amtB1*, *glnK1*, *amtB2*, *glnK2*) (Fig. 1). No signal for *amtB-glnK* transcript was detected in RNA samples extracted from cells grown with ammonium supplementation or yeast extract, suggesting that the *glnK-amtB* gene pairs are not transcribed or, alternatively, their transcripts are targeted for degradation during ammonium rich conditions. In contrast, signals specific for transcripts mapping to both *glnK-amtB* gene pairs were detected in RNA prepared from nitrate grown cells depleted of ammonium. These signals were more intense when RNA was hybridized to the *amtB1-* and *glnK1*-specific probes (probes A and B in Fig. 1) compared to those specific for *amtB2* and *glnK2* (probes C and D) suggesting that *amtB1-glnK1* is the gene pair that is predominantly expressed under nitrate-rich conditions lacking ammonium. The predominant transcript band detected for all four probes (A-D) in the Northern blots was approximately 2000 ribonucleotides corresponding to the size of each *amtB-glnK* gene pair including approximately 350 bp for *glnK* and 1450 bp for *amtB* (Fig. 1). These results, along with the gene organization, suggest that the *amtB-glnK* genes are cotranscribed in pairs. Furthermore, the *amtB-glnK* transcript levels were found to be highly regulated by nitrogen availability with no expression during growth under conditions with ammonium and high level expression during growth with nitrate as the nitrogen source when medium was lacking ammonium. The levels of *amtB-glnK* transcripts agree with our previous study in which GlnK levels were only detected by anti-GlnK immunoblot under nitrate-rich conditions when cells were starved for ammonium (Pedro-Roig et al. 2011). The a*mtB1-glnK1* and *amtB2-glnK2* probes of this study share DNA sequence homology, making it difficult to assess whether both gene pairs are transcribed. However, the differences in intensity of the hybridization signals observed in our Northern blotting analysis suggest *amtB1-glnK1* is the gene pair that is highly expressed during ammonium starvation under nitrate-rich conditions.

### *Haloferax mediterranei pyrE2*-based genetic system established

*Hfx. mediterranei* possesses the complete set of gene homologs of the pyrimidine biosynthesis pathway based on recent genome sequence (Han et al. 2012). Among the biosynthetic homologs are an orotidine-5′-phosphate decarboxylase (*pyrF*) and two orotate phosphoribosyltransferase (*pyrE1* and *pyrE2*) gene homologs. The *pyrE2* gene is routinely used as counterselectable marker during mutant construction in *Hfx. volcanii* (Bitan-Banin et al. 2003; Allers et al. 2004), as it seems to be the physiological orotate phosphoribosyltransferase. During this study, the *pyrE2* gene of *Hfx. mediterranei* was deleted to create parent strain HM26 (Fig. S1, Table 1) for targeted deletion and in-frame Flag-tag insertion of *Hfx. mediterranei* genes by *pyrE2*-based counterselection. The resulting strain HM26 (*ΔpyrE2*) was unable to grow in minimum medium unless supplemented with uracil, in which case the growth rate was equivalent to that of the wild-type strain (Fig. S2, top and middle panels). A lethal 5-FOA concentration for the wild-type strain R4 (750 mg L^−1^) had no inhibitory effect on HM26 (Fig. S2, bottom panel). This finding suggests that HM26 (*ΔpyrE2*) can be used for mutant construction using a method similar to the *pyrE2*-based pop-in pop-out strategy developed for *Hfx. volcanii* (Bitan-Banin et al. 2003; Allers et al. 2004).

### HM26 as parental strain for construction of *glnK* deletion strains

The HM26 strain was used to obtain single (HM26-K1, *ΔglnK1*, and HM26-K2, *ΔglnK2*) and double (HM26-K1K2, *ΔglnK1 ΔglnK2*) *glnK* deletion mutants (Table 1; Fig. S3). With this purpose, deletion cassettes formed by the *glnK1* and *glnK2* flanking regions were cloned into pMH101N to obtain the constructs pMH101N-*ΔglnK1* and pMH101N-*ΔglnK2*. In this case, the typical pop-in pop-out procedure was used in which pop-ins were selected in minimum medium lacking uracil followed by selection of pop-outs in the presence of uracil and 5-FOA. The double deletion was constructed using the *glnK1* single deletion (HM26-K1) as the parental strain. The genotype of all strains at the relevant *glnK* loci was confirmed by Southern blot (Fig. S3).

**Figure 2:**
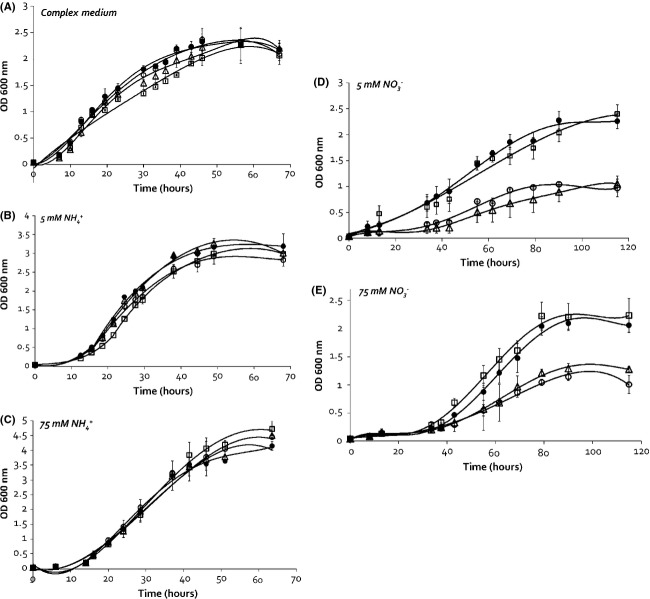
Phenotype analysis of *glnK* deletion mutants. Growth of HM26-K1 (*ΔglnK*_*1*_) (○), HM26-K2 (*ΔglnK*_*2*_) (□), and HM26-K1K2 (*ΔglnK*_*1*_ *ΔglnK*_*2*_) (△) was compared to the parental strain (HM26) (•). Growth curves in (A) yeast extract complex medium and defined media with 0.5% (w/v) glucose and (B) 5 mmol/L NH_4_Cl, (C) 75 mmol/L NH_4_Cl, (D) 5 mmol/L KNO_3_, and (E) 75 mmol/L KNO_3_.

The phenotype of the three *ΔglnK* strains was analyzed in the presence of different nitrogen sources (nitrate, ammonium, and yeast extract) (Fig. 2). No phenotype was observed with ammonium or yeast extract, but a significant growth deficiency was detected in medium supplemented with nitrate for the strains devoid of *glnK1* (HM26-K1 and HM26-K1K2).

### GlnK1/2 are not required for ammonium-induced repression of *amtB1/2* transcript levels

**Figure 3:**
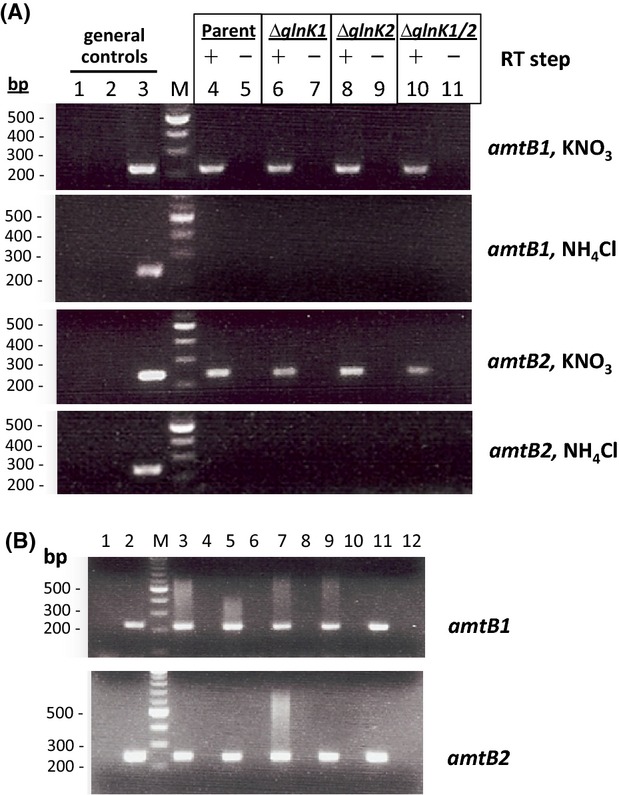
RT-PCR analysis of *amtB-glnK* transcription in *ΔglnK* and *flag:amtB* mutant strains. (A) RT-PCR analysis of *amtB-glnK* transcription in *ΔglnK* strains. First panel: nitrate RNA samples amplified with *amtB1* primers; second panel: ammonium RNA samples amplified with *amtB1* primers; third panel: nitrate RNA samples amplified with *amtB2* primers; fourth panel: ammonium RNA samples amplified with *amtB2* primers. Lanes correspond to the following PCR products: (1) PCR negative control (water instead of template cDNA), (2) RT negative control (cDNA obtained from RT reactions without template RNA), (3) PCR positive control (genomic DNA amplification), (M) *Quick-Load 100 bp DNA Ladder* from *New England Biolabs*, (4 and 5) HM26 RNA sample and corresponding negative control (cDNA obtained from RT reaction with RNA but without retrotranscriptase), (6 and 7) HM26-K1 RNA sample and corresponding negative control, (8 and 9) HM26-K2 RNA sample and corresponding negative control, (10 and 11) HM26-K1K2 RNA sample and corresponding negative control. (B) RT-PCR analysis of the *amtB* transcription in *flag:amtB* strains. The four Flag-tagged strains and HM26 as control were grown in complex medium (OD_600_ of 1) and nitrogen starved for 48 h prior to RNA isolation. Lanes correspond to (1) negative control for the PCR reaction performed in the absence of cDNA, (2) positive control with genomic DNA as PCR template, (M) *Quick-Load 100 bp DNA Ladder* from *New England Biolabs*, (3 and 4) HM26 RT-PCR reaction and negative control in the absence of retrotranscriptase to check for DNA contamination, respectively, the same for (5 and 6) HM26-F1, (7 and 8) HM26-F2, (9 and 10) HM26-F3, (11 and 12) HM26-F4. (A) and (B) RT of the RNA samples was performed with random hexamers and PCR amplification of cDNA with *amtB1* and *amtB2* specific primers (RT-Amt1For/RT-Amt1Rev and RT-Amt2For/RT-Amt2Rev, respectively). PCR amplification products of the cDNA separated by 1.8% (w/v) agarose gel electrophoresis and stained with ethidium bromide are presented.

The *glnK* deletion mutants were used to determine whether GlnK proteins control their own transcription (i.e., the transcription of the *amtB-glnK* gene pair). With this purpose, parent and mutant strains were grown with two different nitrogen sources (nitrate or ammonium), and total RNA isolated from these cells was analyzed by end-point RT-PCR using primer pairs specific for *amtB1* and *amtB2*. The cDNA fragments amplified from total RNA, using *amtB1* and *amtB2* primer pairs, were 225 and 244 bp, respectively. The RT-PCR patterns of the single (HM26-K1, HM26-K2) and double (HM26-K1K2) *glnK* mutants were compared to the HM26 parental strain by gel electrophoresis (Fig. 3A). Consistently with the previous Northern blot results, *amtB1/2*-specific transcripts were detected by RT-PCR when cells were grown on nitrate but not when ammonium was supplied as the nitrogen source. This same pattern of RT-PCR product was conserved in parent and *glnK1/2* deletion strains suggesting that GlnK proteins do not influence their own transcription or at least are not required for the ammonium-induced repression of *glnK-amtB* transcript levels. When comparing *amtB1* to *amtB*2, the signals detected for each transcript by end-point RT-PCR were equivalent while those obtained by Northern blotting were significantly different (with hybridization signals much higher for *glnK1*-*amtB1* than for *glnK2*-*amtB2*). In contrast to RNA-seq data (Garber et al. 2011; Lee et al. 2011), which can be used for quantitative comparison of transcript levels encoded by different genes, RT-PCR and Northern blot techniques are notoriously limited in use for quantitative comparison between genes. However, our findings demonstrate that *glnK-amtB* are cotranscribed, and that both gene pairs are expressed in *Hfx. mediterranei* when grown on nitrate but not ammonium.

### *pyrE2*-based counterselection for strains encoding Flag-tagged AmtB1/2 fusions

GlnK PII homologs are typically encoded by genes adjacent to high-affinity ammonium transporter (*amtB*) genes (Thomas et al. 2000). Consistent with this genetic linkage, GlnK proteins interact with and regulate AmtB transporters in bacteria (Coutts et al. 2002; Strösser et al. 2004; Zhang et al. 2006; Huergo et al. 2010). Nevertheless, no experimental proof exists for such an interaction in archaea. In this work, a GlnK immunoprecipitation strategy using Flag-tagged AmtB proteins was performed with this purpose.

The *pyrE2*-based counterselection procedure is frequently applied to obtain targeted genomic modifications. As described above, both *glnK* genes were readily deleted from *Hfx. mediterranei* genome using this approach. Thus, the same method was applied to introduce a Flag-tag coding sequence into the genomic copies of the ammonium transporter genes (*amtB1/2*) of this organism. Four mutants were obtained and named HM26-F1 to HM26-F4, each one harboring a modified gene encoding a Flag-tag fused to the N- or C-terminus of AmtB1/2 (Table 1; Fig. S4).

### Flag-tagged *amtB1/2* genes are transcribed

Transcript levels of the Flag-tagged *amtB1/2* genes were analyzed in HM26-F1 to F4 strains by RT-PCR to ensure that the introduction of the foreign sequence did not prevent gene expression. Cells were grown in complex medium and nitrogen starved to induce *amtB1/2* expression prior to RNA isolation. As was done previously with the *glnK* deletion mutants, cDNA fragments specific for *amtB1* (225 bp) and *amtB2* (244 bp) transcripts were generated by RT-PCR from parent and Flag-tag fusion strains. With this approach, cDNA specific for *amtB1/2* was detected in all strains examined (Fig. 3B). This result indicated that the *amtB1/2* gene was transcribed in HM26-F1 to F4 strains and that insertion of the Flag-tag coding sequence did not prevent its expression.

### Localization of AmtB1-Flag in *Hfx. mediterranei* membranes

**Figure 4:**
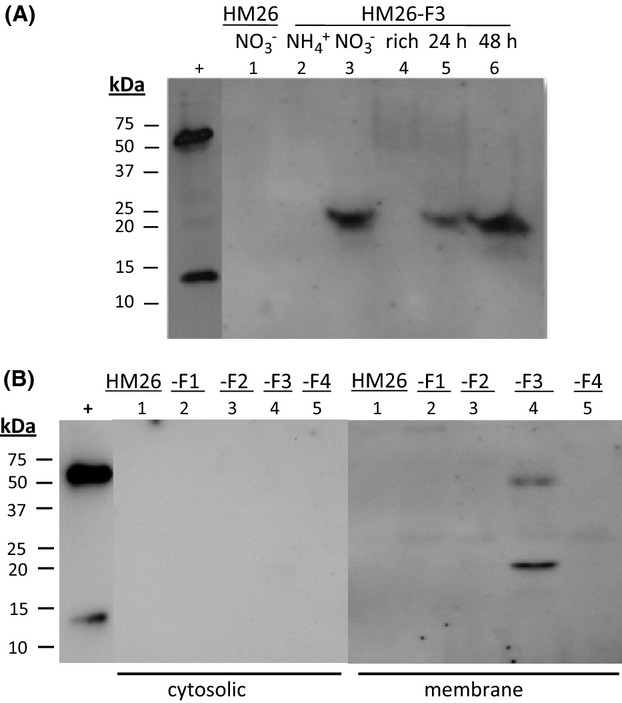
Anti-Flag immunoblot of *Hfx. mediterranei* strains with Flag-tag coding sequence insertions in *amtB1/2*. (A) Anti-Flag immunoblot of whole cell lysate of HM26-F3 (*amtB1-flag*) strain grown with different sources of nitrogen. Lysate was obtained by boiling whole cells in reducing SDS-PAGE loading buffer prior to separation of proteins by 12% SDS-PAGE (with cells in OD_600_ per lane of 0.325 units for HM26-F3 and HM26 and 0.065 units for the positive control [+, *Hfx. volcanii* H26-pJAM947 expressing Flag-SAMP1]). HM26 was grown in 75 mmol/L KNO_3_ (1) and HM26-F3 was grown in (2) 75 mmol/L NH_4_Cl, (3) 75 mmol/L KNO_3_, and (4) complex medium. HM26-F3 strain was also grown in complex medium and nitrogen starved for (5) 24 h and (6) 48 h. (B) Cytoplasmic and membrane fractions of *Hfx. mediterranei* strains with Flag-tag coding sequence insertions in *amtB1/2*. All four Flag-tagged strains (HM26-F1 to F4) and parent HM26 (as negative control) were grown in complex medium (to an OD_600_ of 1) and transferred to minimal medium with 0.5% (w/v) glucose and no nitrogen source for 48 h. Protein (50 μg) from membrane or cytoplasmic fractions was separated by 12% SDS-PAGE and analyzed by anti-Flag immunoblotting. Lanes correspond to (+) Flag-SAMP1 from *Hfx. volcanii* as positive control, (1) HM26 strain, (2) HM26-F1 (*flag-amtB1*), (3) HM26-F2 (*flag-amtB2*), (4) HM26-F3 (*amtB1-flag*), (5) HM26-F4 (*amtB2-flag*). For all samples, proteins transferred to membranes were analyzed by staining with Ponceau Red to confirm equal protein loading prior to analysis by anti-Flag immunoblot. Migration of *Kaleidoscope Molecular Weight Markers* (*Bio-Rad*) indicated on the left.

The levels of the Flag-tagged AmtB1/2 proteins produced by the recombinant *Hfx. mediterranei* strains (HM26-F1 to F4) were analyzed by anti-Flag immunoblot. To identify conditions for detection of the tagged AmtB1/2, recombinant and parent HM26 strains were grown under different nitrogen conditions (nitrate, ammonium, and yeast extract) and starved for nitrogen. With this approach, a Flag-specific protein band was detected in HM26-F3 (*amtB1-flag*) but not in parent HM26 (Fig. 4A) or the other recombinant strains (data not shown). The apparent molecular mass of the immunoreactive band was 20 kDa, which was clearly smaller than the mass expected for a Flag-tagged AmtB1 (50 kDa). However, the levels of this 20 kDa protein appeared regulated by nitrogen with the Flag-specific protein detected only during growth with nitrate or after nitrogen starvation conditions. This nitrogen-regulated expression was in good agreement with the profiles observed for *glnK1-amtB1* transcript (see above) and GlnK protein (Pedro-Roig et al. 2011). The results suggested that AmtB1 could be produced in *Hfx. mediterranei* with a C-terminal Flag tag but was predominantly in a cleaved form.

High-affinity ammonium transporters have been described as multipass membrane proteins in many organisms, but no proof existed for haloarchaea, as AmtB proteins had not been analyzed in this family of microorganisms until now. In order to check whether the AmtB1/2 proteins of *Hfx. mediterranei* were associated in the membranes, cell lysate was separated into membrane and cytoplasmic fractions and analyzed by anti-Flag immunoblot. Prior to lysis, cells were submitted to nitrogen starvation for 48 h, as transcription of *amtB1* and *amtB2* had been detected by RT-PCR under these conditions. Membrane and cytoplasmic fractions of the starved cells were analyzed by anti-Flag immunoblotting to determine AmtB subcellular localization. As can be appreciated in Figure 4B, none of the strains showed any signal in the cytoplasmic fraction, but two anti-Flag immunoreactive bands were detected in HM26-F3 membranes. One of those signals presented an apparent molecular mass around 50 kDa, which corresponds to the entire AmtB1-Flag chain whereas the other was at 20 kDa, matching the protein band detected in cell lysate. The absence of any anti-Flag immunoreactive bands in parent HM26 indicated that the signals were specific to HM26-F3 and that the signal of 20 kDa must correspond to a cleaved form of AmtB1-Flag (C-terminal fragment). The 20 kDa molecular mass suggests this truncated form of AmtB1-Flag may start before the first amino acid of its last predicted transmembrane helix (TMH) (see Fig. S5). The prediction of at least one TMH in this C-terminal AmtB1-Flag fragment would explain why the 20kDa signal appears in the membrane fractions.

### GlnK proteins are coimmunoprecipitated with AmtB1-Flag

Given that only AmtB1-Flag proteins were successfully incorporated into *Hfx. mediterranei* membranes whereas the other Amt variants failed, strain HM26-F3 (*amtB1:flag*) was used for GlnK coimmunoprecipitation studies (co-IP). Strains HM26-F3 and HM26 (as negative control) were grown in complex medium and nitrogen starved for 48 h to induce *amtB1* expression. Finally, ammonium (150 mmol/L NH_4_Cl) was added and cells were incubated for 1 h prior to harvesting, to stimulate association of GlnK with AmtB1, given that AmtB-GlnK complexes are detected after ammonium shock in bacteria (Coutts et al. 2002). Membrane fractions were isolated and, before first proceeding to co-IP, the fractions were tested for purity by absence of isocitrate dehydrogenase (ICDH) activity, the activity of which was readily detected in the cytoplasmic fractions of these cells (see Fig. S6). Once the purity was confirmed, GlnK presence in the resuspended membrane fractions was determined by anti-GlnK immunoblot (Fig. S7). Two reactive bands of slightly different electrophoretic mobility were detected (Fig. S7), which can be attributed to GlnK1 (lower molecular mass) and GlnK2 (higher molecular mass) (Pedro-Roig et al. 2013b).

**Figure 5:**
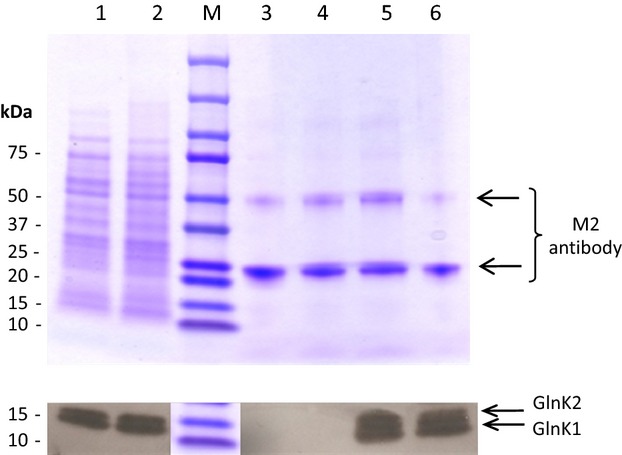
Anti-GlnK immunoblot of the AmtB1-Flag immuno-precipitation experiment. HM26 and HM26-F3 strains were grown in complex medium and nitrogen starved for 48 h prior to a 150 mmol/L NH_4_Cl shock for 1 h. Solubilized membrane fractions were anti-Flag immunoprecipitated and the elution fractions were separated in a 15% SDS-PAGE and anti-GlnK immunoblotted. The upper panel shows a Coomassie Blue staining, the lower panel shows the anti-GlnK immunoblot. Membrane protein (15 μg) was loaded in lanes 1 (HM26 strain) and 2 (HM26-F3 strain). (M) *Kaleidoscope Molecular Weight Markers* (*Bio-Rad*), (lanes 3 and 4) HM26 first and second IP elution fractions and (lanes 5 and 6) HM26-F3 first and second IP elution fractions. Elution fractions (5 μL) were loaded in the Coomassie stained gel whereas 30 μL were loaded in the immunoblotted gel.

Next, we examined whether GlnK in the membrane was associated with AmtB1-Flag. For this analysis, membrane fractions were solubilized in 2% (v/v) Triton X-100, immunoprecipitated with anti-Flag M2 affinity gel beads, and assayed for the presence of GlnK by immunoblotting with anti-GlnK antibody (see Experimental Procedures for details) (Fig. 5). GlnK was present in the solubilized membrane fractions of both HM26 and HM26-F3 strains (Fig. 5, lanes 1 and 2). However, only α-Flag IP elution fractions of HM26-F3 (and not HM26) showed GlnK reactive bands (lanes 5 and 6 compared to 3 and 4). This strain specific co-IP (dependant on synthesis of AmtB1-Flag) provided strong evidence that stable specific interactions exist between AmtB1 and GlnK. Furthermore, based on detection of two distinct GlnK-specific bands, these findings also suggested that AmtB1 interacts with both GlnK1 and GlnK2. Alternatively, a subset of GlnK protein associated with AmtB1 is covalently modified (e.g., uridylylated, cleaved). However, in *E. coli*, only the deuridylylated form of GlnK appears to be sequestered and inhibit transport of ammonium by AmtB (Javelle et al. 2004).

### AmtB-GlnK interaction depends on the presence of ammonium in the medium

**Figure 6:**
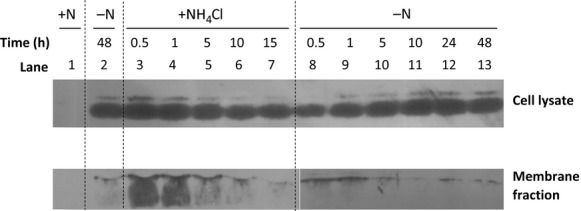
Amt-GlnK interaction reversibility assay. Membrane fractions and whole cell lysates from HM26 cells were anti-GlnK immunoblotted for comparison. Cells were analyzed under the following conditions: growth to OD_600_ 1.0 in complex medium (lane 1); 48-h nitrogen starvation (lane 2); ammonium shock (150 mmol/L NH_4_Cl) for 0.5, 1, 5, 10, and 15 h (lanes 3–7); cells transferred again to nitrogen devoid medium for 0.5, 1, 5, 10, 24, and 48 h (lanes 8–13).

To find out whether the association of GlnK with AmtB in the membranes is reversible, cells were grown in complex medium, nitrogen starved for 48 h, and exposed to an ammonium shock as performed for the co-IP experiment, only to be transferred again to a medium devoid of nitrogen and monitored for GlnK-AmtB interactions over time. These reversibility assays were carried out using the HM26 parental strain, as evidence for specificity of AmtB1-GlnK interaction was already demonstrated (see above). GlnK presence in whole cell lysate and membrane fractions was analyzed by anti-GlnK immunoblot (Fig. 6), and membrane fraction purity was confirmed by the absence of ICDH activity as before (Supporting Material Fig. S8).

With this approach, GlnK association with AmtB1 in cell membranes was found to be rapid, reversible, and dependent on nitrogen source (Fig. 6). GlnK was not present in cells grown in complex medium to early stationary phase (OD_600_ 1) (lane 1). However, after cells were starved for nitrogen (48 h), GlnK was readily detected in cell lysate but not in membrane fractions (lane 2). After ammonium shock (lanes 3–7), GlnK rapidly accumulated in the membrane (with this association predominant within the first 30 min after addition of NH_4_Cl to nitrogen starved cells). GlnK protein was found to no longer be associated at high levels with membrane fractions after further incubation with ammonium (lanes 6–7) or after removal of the ammonium source (lanes 8–13). By contrast to membrane fractions, the overall levels of GlnK protein in the cell lysate appeared relatively constant with a slight decrease in protein levels after ammonium shock (lanes 5–7) that was relieved by nitrogen starvation (lanes 10–13). Overall, this time course shows that the GlnK-AmtB membrane protein interaction is reversible and depends on the presence of ammonium in the medium.

## Discussion

GlnK proteins are PII homologs encoded adjacent to the gene for the ammonium transporter AmtB, and this nearly invariable linkage was thought to imply a functional and widely distributed relationship between the regulator and the transporter (Thomas et al. 2000). In 1998, GlnK was described as a negative regulator of AmtB activity in *Azospirillum brasilense* based on two experimental observations: on the one hand, a mutation in *glnZ* (*glnK* homolog) doubled the rate of ammonium uptake by AmtB and, on the other hand, this ammonium uptake was reduced by the presence of additional copies of *glnZ* (de Zamaroczy 1998). Interaction of GlnK and AmtB was later described (Coutts et al. 2002), and three-dimensional structures of this interaction are solved (Conroy et al. 2007; Gruswitz et al. 2007). The complexes crystallize as the association of one AmtB trimer and one GlnK trimer, showing structural coevolution. The physiological role of the AmtB-GlnK interaction is to modulate the activity of the transporter to optimize ammonium uptake under different growth conditions (Coutts et al. 2002). AmtB has been suggested to be a signal transduction protein, and the AmtB-GlnK system to provide a means not only for responding to the presence of the extracellular substrate, but also for sensing how much ammonium is entering the cell (Coutts et al. 2002).

Almost all members of the *Bacteria* and *Archaea* Domains have their *amtB* genes linked to *glnK*, suggesting that this is a conserved interaction. The interaction of GlnK-AmtB has been demonstrated in bacteria by analyzing the presence of PII proteins in membrane fractions before and after an ammonium pulse and comparing the results to *amtB* deficient mutants by immunoblot (Coutts et al. 2002; Detsch and Stülke 2003; Strösser et al. 2004; Huergo et al. 2006, 2010; Zhang et al. 2006; Tremblay et al. 2007). Nevertheless, a GlnK-AmtB interaction had not been previously identified in an organism of the *Archaea* Domain nor demonstrated in any organism using an immunoprecipitation strategy as the one described here. A theoretical model of a complex structure for GlnK and AmtB proteins of the archaeon *Archaeoglobus fulgidus* was published (Andrade et al. 2005), although no experimental proof was provided.

In this work, GlnK was found to associate with AmtB in membrane fractions of *Hfx. mediterranei* after ammonium shock using a coimmunoprecipitation (co-IP) strategy. In order obtain an *Hfx. mediterranei* strain that synthesized AmtB1-Flag for co-IP, genomic insertions mutants were constructed using a gene replacement system based on homologous recombination and the *pyrE2* gene as counterselectable marker. Similarly to *Hfx. volcanii* (Bitan-Banin et al. 2003; Allers et al. 2004), a *pyrE2*-based approach proved useful for genetic modification of *Hfx. mediterranei*. During the course of this work, a gene deletion system was developed for *Hfx. mediterranei* using *pyrF* as counterselectable marker (Liu et al. 2011). Similarly to the *pyrE2* gene used in our system, *pyrF* is required for *de novo* pyrimidine biosynthesis and confers uracil auxotrophy in strains with mutations in this gene.

Four Amt Flag-tagged insertion mutants were constructed by placing a Flag-tag in either the N- or C-terminus of one of the two *amtB1/2* homologues. Only in the C-terminal Flag-tagged AmtB1 strain (HM26-F3, *amtB1-flag*) could anti-Flag signals be detected, even though transcription of all four gene variants was successful. AmtB1-Flag was present in the membrane, which is the first experimental proof of AmtB1 proteins being membrane proteins in haloarchaea. Failure to detect a signal for strains HM26-F1 (*flag-amtB1*) and HM26-F2 (*flag-amtB2*) may be attributed to proteolytic cleavage of the Flag-AmtB in due to interruption of a putative N-terminal signal peptide required for proper insertion of AmtB into the membrane or cleavage of the signal peptide (and associated Flag-tag) during AmtB maturation. In Gram-negative bacteria such as *E. coli*, AmtB is encoded as a preprotein with an N-terminal signal peptide that is cleaved to produce the mature transporter composed of 11 transmembrane helices and a cytosolic C-terminal domain (Thomas et al. 2000; Thornton et al. 2006). In comparison, the N-terminus of the *Hfx. mediterranei* AmtB polypeptide is somewhat shorter and is not predicted to include a signal peptide for either the general secretory (Sec) or twin-arginine translocation (Tat) pathways (based on analyses using SignalP, PRED-SIGNAL, TMHMM, and TATFind). Another possible explanation for the failure of strains HM26-F1 and -F2 could be the negative-charged residues of the Flag-tag that are electrostatically repulsed from the membrane phospholipids. The C-terminal region of AmtB1 is 66 amino acids longer than that of AmtB2, which could be related to the finding that the C-terminal Flag-tagged AmtB1 was successfully incorporated into the membrane, whereas the same version of AmtB2 (HM26-F4, *amtB2-flag*) was not.

Interestingly, fusion of a Flag-tag to the C-terminus of AmtB1 did not disrupt its regulated binding to GlnK in *Hfx. mediterranei*. By contrast, deletions and site-directed modifications in residues within the C-terminal region (CTR) of the *E. coli* AmtB abolish its association with GlnK (Severi et al. 2007). The disruption in protein–protein interactions of *E. coli* AmtB-GlnK can be explained by X-ray crystal structures of this complex, where residues in the C-terminal region (CTR) of AmtB are noncovalently bonded to GlnK (Conroy et al. 2007; Gruswitz et al. 2007). Similarly to *E. coli* AmtB, the *Hfx. mediterranei* AmtB1 is likely to adopt an 11 transmembrane helix topology based on TMHMM (Server v. 2.0)-prediction of transmembrane helices. Furthermore, bacterial (*E. coli*) and archaeal (*Archaeoglobus fulgidus*) AmtB proteins adopt a similar fold in X-ray crystal structures (Andrade et al. 2005; Conroy et al. 2007; Gruswitz et al. 2007). However, *Hfx. mediterranei* AmtB1 has a putative cytosolic C-terminal domain that is ∼90 residues longer than that of *E. coli* AmtB and, thus, is likely to accommodate modifications that do not perturb GlnK binding such as a Flag-tag fusion.

The organization of the *Hfx. mediterranei* genome suggests AmtB1-GlnK1 and AmtB2-GlnK2 interactions are specific; however, the coIP experiments reveal possible cross-reactive interactions among these paralogs. In particular, we found that AmtB1 bound two distinct GlnK isoforms with immunoblot signals suggesting a relatively equimolar ratio of GlnK1 and GlnK2 bound AmtB1. The primary amino acid sequences of GlnK1 and GlnK2 share 86% identity and 90% similarity. Thus, GlnK1/2 appear similar enough for the proteins to have interchangeable activities, consistent with their activating glutamine synthetase in vitro in an equivalent way (Pedro-Roig et al. 2013b). However, it is also possible that AmtB1 binds GlnK1 isoforms that are modified posttranslationally. Regarding AmtB2, no data about its interaction with GlnK1/2 could be obtained with this experimental design. By homology, it is likely that AmtB2 interacts with both GlnK1 and GlnK2, however, in some organisms it has been described that not all the Amt homologues are regulated by GlnK binding, for example, in *Corynebacterium glutamicum* GlnK binds AmtB, but not AmtA (Strösser et al. 2004). A similar situation exists in *Rhodobacter capsulatus*, in which second Amt homologue (AmtY) is not blocked by PII proteins (Tremblay et al. 2007).

GlnK-AmtB complex formation enables the cell to block the entrance of ammonium when this nutrient is not required or the energetic balance of the cell does not favor nitrogen assimilation. Therefore, GlnK-AmtB interaction must be reversible, so it can be disrupted when environmental conditions change (Coutts et al. 2002; Maier et al. 2011). To determine whether this was the case in *Hfx. mediterranei*, HM26 strain was used to check GlnK presence or absence in the membrane following a high–low nitrogen availability cycle. Results showed that the total amount of GlnK in the cell increased under starvation and decreased when ammonium was supplied. GlnK presence in the membrane also depended on the ammonium availability. One consequence of the Amt-mediated GlnK sequestration would be the depletion of the cytoplasmic GlnK pool, which may help amplify the regulatory properties of GlnK with respect to any cytoplasmic targets (Coutts et al. 2002).

In this study, the levels of the *Hfx. mediterranei amtB-glnK* transcripts were analyzed by Northern blotting and RT-PCR and found to be highly regulated by nitrogen availability with no transcript molecules detected under conditions with ammonium and high levels of transcript detected when nitrate served as the nitrogen source instead of ammonium. This finding points out that *glnK* expression is not necessary during growth with the preferred nitrogen source (ammonium) (Martínez-Espinosa et al. 2007) and agrees with our previous analysis of GlnK expression regulated by nitrogen at the protein level (Pedro-Roig et al. 2011). In Gram-negative bacteria such as *E. coli* and *Pseudomonas putida*, *glnK-amtB* is similarly regulated by nitrogen availability with expression silent in the presence of ammonium and high under conditions of ammonium starvation (Atkinson et al. 2002; Hervás et al. 2009). In these bacteria, *glnK is* transcriptionally activated at a σ^N^ (σ^54^)-dependent promoter by the phosphorylated form of the enhancer-binding protein NtrC (GlnG, NRI), with the levels of phosphorylated NtrC (NtrC-P) controlled by the bifunctional histidine kinase NtrB (GlnL, NRII) (Atkinson et al. 2002; Hervás et al. 2009). NtrC-P activates *glnK* transcription by binding upstream of the σ^N^-dependent promoter (with RNA polymerase-mediated open complex formation requiring integration host factor [IHF] in *P. putida*) (Atkinson et al. 2002; Hervás et al. 2009). While σ factors are an essential component of the bacterial RNA polymerase, the basal transcription machinery of Archaea is instead strikingly similar to the core components of eukaryotic RNA polymerase (RNAP) II (Werner and Grohmann 2011). Archaea do not rely upon sigma factors and instead use promoters consisting of a TATA box 25 bp upstream of the transcription start site that forms a ternary complex with TATA box-binding protein (TBP) and transcription factor TFIIB (TFB) homologues (Werner and Grohmann 2011). While two-component signal transduction systems are conserved in Archaea (Eichler and Maupin-Furlow 2013) and the basal transcription machinery of Archaea often associates with homologs of bacterial transcriptional regulators (Bell and Jackson 1998), close homologs of NtrC and NtrB are not identified in *Hfx. mediterranei* suggesting a novel mechanism that mediates the nitrogen regulation of *amtB-glnK* transcript levels in this archaeon.

## Supporting Information

**Table S1:** Primers used in this study.

**Figure S1**: Southern blot analysis of HM26 (*ΔpyrE2*).

**Figure S2**: Comparison of *Hfx. mediterranei* wild-type (R4) and HM26 strains phenotype.

**Figure S3:** Southern blot analysis of *glnK* deletion strains.

**Figure S4:** Southern Blot analysis of the *flag:amt* knockin strains.

**Figure S5**: *Hfx*. *mediterranei* Amt topology predictions.

**Figure S6:** Membrane fraction purity control of IP samples.

**Figure S7**: Anti-GlnK immunoblot of HM26 and HM26-F3 membrane fractions.

**Figure S8:** Membrane fraction purity control of the reversibility assay samples.

**Figure S9**: Alignment of *Hfx. mediterranei* amt genes showing the primers used in the RT-PCR analyses.
