# Supplementary material for: Nitrogen regulation of protein–protein interactions and transcript levels of GlnK PII regulator and AmtB ammonium transporter homologs in Archaea
Source: Microbiologyopen. 2013 Aug 28;2(5):826–40. doi: 10.1002/mbo3.120 (PMC3831643; doi:10.1002/mbo3.120)
Supplement: Supplementary file 1 [file mbo30002-0826-SD1.docx]

**Table S1**: Primers used in this study.

| **Primer name** | **Sequence (5’-3’)** | **Purpose** |
| --- | --- | --- |
| glnK1-mRNA-1F | gcgagactgagaccggaaacgacgg | Northern blot |
| glnK1-mRNA-1R | tcagactgctggactcccctccttgc |  |
| glnK2-mRNA-2F | gacgctgacctgccgaacgacgg-3’ |  |
| glnK2-mRNA-2R | ccggtccgaacctgaatcgcgtt |  |
| amt1-mRNA-3F | ggcgctgtcccgcacgtcacg |  |
| amt1-mRNA-3R | agtccggacatcgttcccaccgtcagt |  |
| amt2-mRNA-4F | gtctctacgcacccgacgcctcgg |  |
| amt2-mRNA-4R | cgtaggcgaacgagccgagggtcac |  |
| ProbeF-rev | CACCACCCACTTGTTCGCGTCGC | Southern blot |
| RTAmt1-For | GTTCTCGTACATCGGCGACTCCGG | RT-PCR |
| RTAmt1-Rev | AGCGCGGCACCGAGATAGC |  |
| RTAmt2-For | TGTGGGTCCTCACCGTCACGTT |  |
| RTAmt2-Rev | GCGTCGGGTGCGTAGAGACC |  |
| glnK1-1F | tcattccggtgttcgccgtcgg | *glnK_1_* deletion cassette construction |
| glnK1-1R | tgctcagactgcctcgctcattggttttcacctccg |  |
| glnK1-2F | caatgagcgaggcagtctgagcacaacaacgaatg |  |
| glnK1-2R | agccggcgtgcatgaagaagatg |  |
| glnK2-3F | ccgccatcgccgacgacatcg | *glnK_2_* deletion cassette construction |
| glnK2-3R | agaccgcgtcgtcactcatggcttattcgtcctc |  |
| glnK2-4F | gccatgagtgacgacgcggtctgataaggactgttg |  |
| glnK2-4R | cagcgacgtgaactccgacatgt |  |
| pyrE2-5F | cgccgggttcggtctcgccc | *pyrE2* deletion cassette construction |
| pyrE2-5R | ttagttgtcggcgttcgccatcttgtttcaacaggaac |  |
| pyrE2-6F | aagatggcgaacgccgacaactaatacacgcttgtg |  |
| pyrE2-6R | gtacgcgaggcgcgcgccga |  |
| Flag-1F | TAGCTCGAGGGTGCGGACCTCATTATTAC | *flag:amt_1_* suicide plasmid construction |
| Flag-1R | ATGGATCCCACATATGGACGACGGTCG |  |
| Flaginv-5F | GACAAGGGTACCATGCAGCCGGGGTTCG |  |
| Flaginv-5R | GTCGTCGTCCTTGTAGTCCATGAAGAAGATGAGGAACGAGACGAC |  |
| Flag-2F | ATATAAGCTTATCGCGGAGGCCGCCCACACG | *flag:amt_2_* suicide plasmid construction |
| Flag-2R | ATGGATCCAAAGTCGTGGAAGCCGAGACCGTT |  |
| Flaginv-6F | CGACAAGGGTACCATGCTGACCGCAC |  |
| Flaginv-6R | TCGTCGTCCTTGTAGTCCATACGATTCCTCCGATATGC |  |
| Flag-3F | ATATAAGCTTCCCGCGTACCGCTGGACC | *amt_1_:flag* suicide plasmid construction |
| Flag-3R | ATGGATCCGTTTACTAATACGAATGGCTCGGGCA |  |
| Flaginv-7F | 5’-GACGACGACAAGGGATGAGCGAGACTGAGACCGG |  |
| Flaginv-7R | GTCCTTGTAGTCGGTACCTTGGTTTTCACCTCCGTCGACTG |  |
| Flag-4F | ATATAAGCTTTCTACGTCGCAAACGGC | *amt_2_:flag* suicide plasmid construction |
| Flag-4R | ATGGATCCGGATTTCGACATTCACCA |  |
| Flaginv-8F | GACGACGACAAGTAAGCCATGAGTGACGCTGACC |  |
| Flaginv-8R | GTCCTTGTAGTCGGTACCTTCGTCCTCCTGTGTCGT |  |
