# Supplementary material for: Nitrogen regulation of protein–protein interactions and transcript levels of GlnK PII regulator and AmtB ammonium transporter homologs in Archaea
Source: Microbiologyopen. 2013 Aug 28;2(5):826–40. doi: 10.1002/mbo3.120 (PMC3831643; doi:10.1002/mbo3.120)
Supplement: Supplementary file 2 [file mbo30002-0826-SD2.docx]

**Supplemental Figures**

**A**

**B**

| **Strain** | **Genomic DNA** | **Fragment size (bp)** |
| --- | --- | --- |
| **Wild-type** | Wild type (R4) | 1397 |
| **Pop-in** | Wild type +  pMH101N-Δ*pyrE2* | 1397, 1770 |
| **Pop-out** | Wild type +  pMH101N-Δ*pyrE2* | 2091 |

**Wild-type (R4)**

3’

5’

*1397 bp*

**PCR 5**

**PCR 6**

*pyrE2*

5’

**Pop-in B**

*1397 pb*

*1770 pb*

3’

5’

*2091 pb*

**Pop-out**

**(HM26, Δ*pyrE2*)**

3’

Δ*pyrE2*


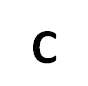


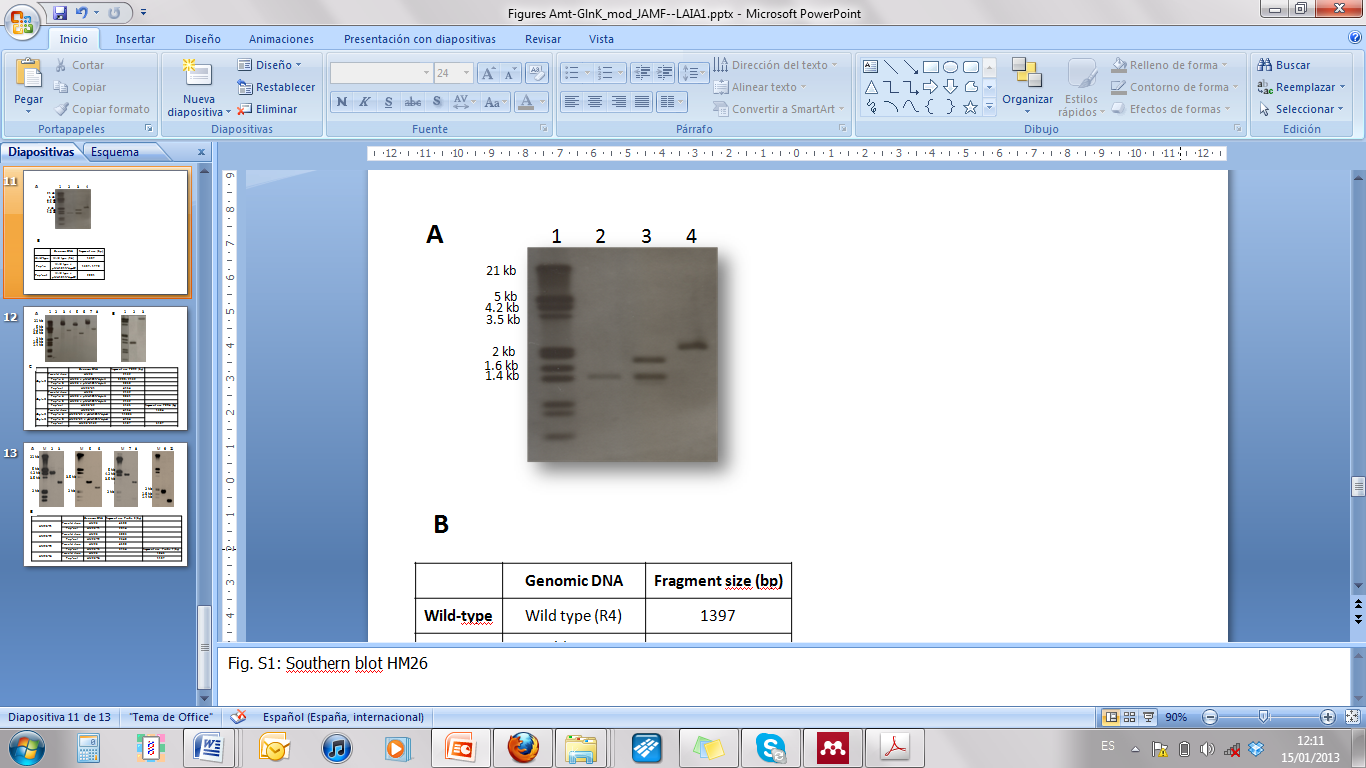


**Figure S1**: Southern blot analysis of HM26 (Δ*pyrE2*). (**A**) DNA (3 μg) was *Sau*96I digested, separated in a 1 % (w/v) agarose gel and probed against the 536-bp DIG-labeled PCR5 fragment amplified with primers pyrE2-5F and pyrE2-5R (Table 2). Lanes correspond to (1) DIG-labeled markers, (2) wild-type *Hfx. mediterranei*, (3) pMH101N-Δ*pyrE2* pop-in strain and (4) HM26 strain. (**B**) Theoretical sizes of the *Sau*96I digested DNA fragments that would hybridize with the probe in each genomic organization. (**C**) Organization of the *pyrE2* genomic region in the different stages of HM26 strain construction. Vertical arrows indicate *Sau*96I restriction sites; blue double-ended arrows indicate DNA fragments from the *Sau*96I digestion that hybridize when PCR5 is used as probe. Genomic elements sizes are not to scale.


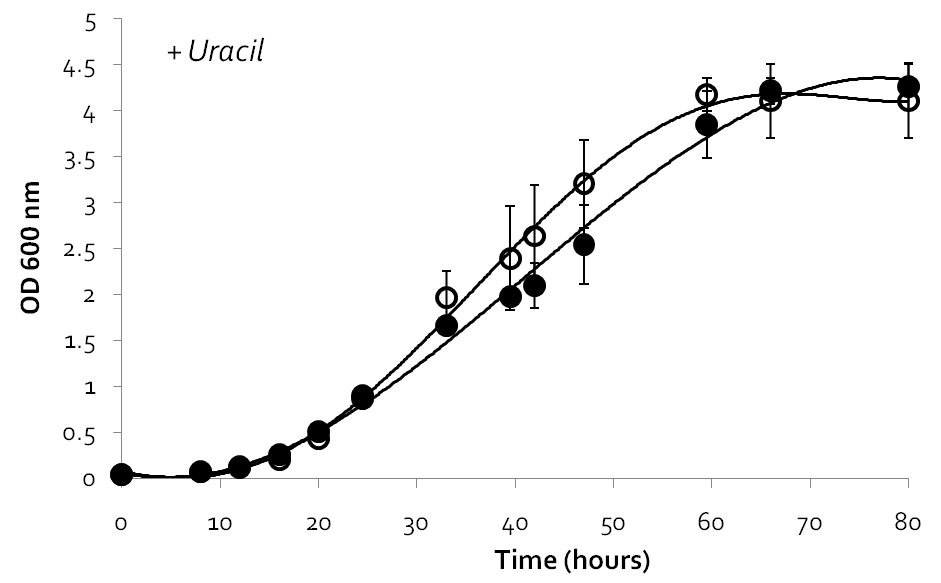

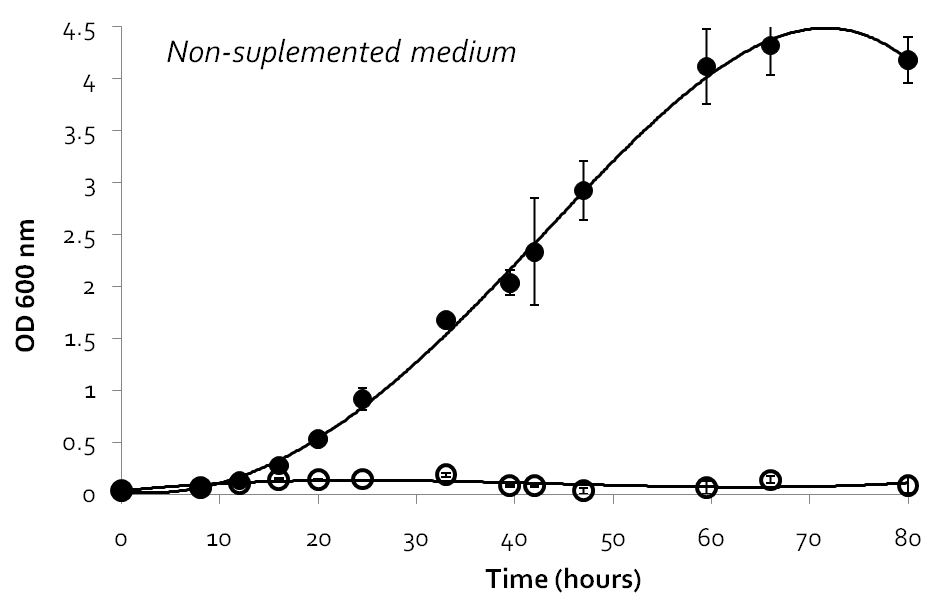


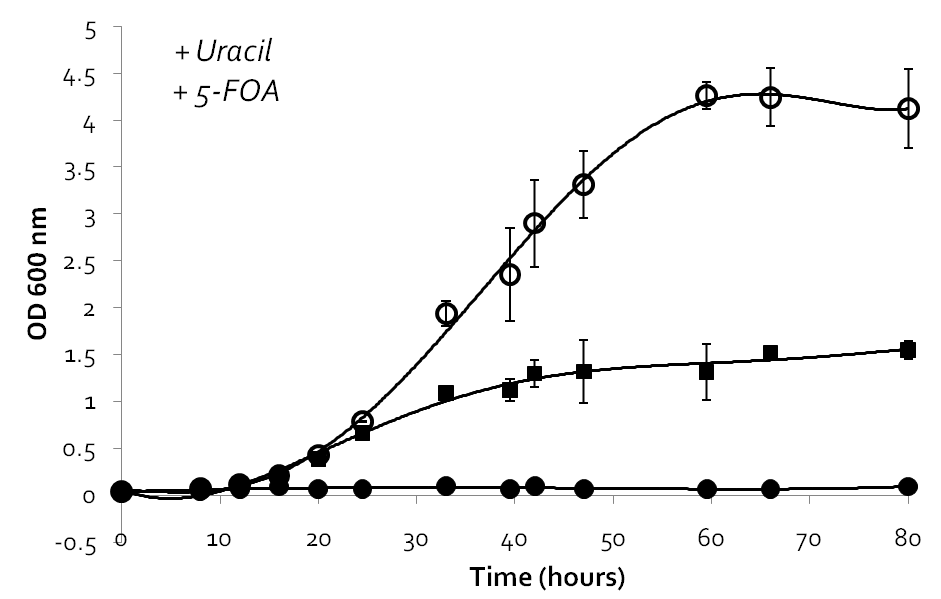


**A**

**B**

**C**

**Figure S2**: Comparison of *Hfx. mediterranei* wild-type (R4) and HM26 strains phenotype. Cells were grown in minimum medium with 0.5 % (w/v) glucose and 75 mM NH_4_Cl supplemented with uracil or uracil plus 5-FOA as indicated. ● and ⭘ represent wild-type and HM26 strains respectively. In (**C**), 750 mg l^-1^ 5-FOA was added except for ■, which represents the wild-type strain growth curve in the presence of 150 mg l^-1^ 5-FOA.


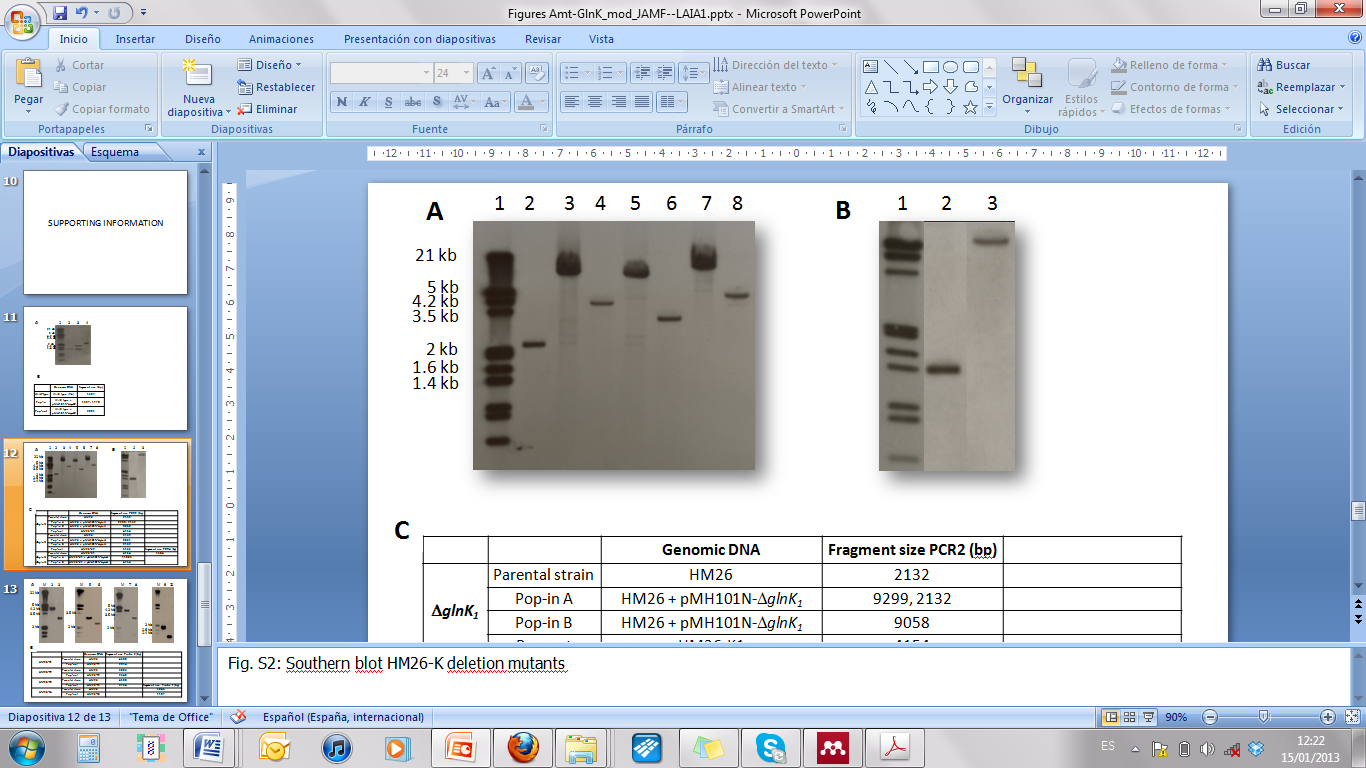


**C**

|  | **Strain** | **Genomic DNA** | **Fragment size PCR2 (bp)** | **Fragment size PCR4 (bp)** |
| --- | --- | --- | --- | --- |
| **Δ*glnK_1_*** | Parent | HM26 | 2132 | - |
|  | Pop-in A | HM26 + pMH101N-Δ*glnK_1_* | 9299, 2132 | - |
|  | Pop-in B | HM26 + pMH101N-Δ*glnK_1_* | 9058 | - |
|  | Pop-out | HM26-K1 | 4154 | - |
| **Δ*glnK_2_*** | Parent | HM26 | 2132 | - |
|  | Pop-in A | HM26 + pMH101N-Δ*glnK_2_* | 9061 | - |
|  | Pop-in B | HM26 + pMH101N-Δ*glnK_2_* | 2132 | - |
|  | Pop-out | HM26-K2 | 3165 | - |
| **Δ*glnK_1_***  **Δ*glnK_2_*** | Parental strain | HM26-K1 | 4154 | 1384 |
|  | Pop-in A | HM26-K1 + pMH101N-Δ*glnK_2_* | 11083 | - |
|  | Pop-in B | HM26-K1 + pMH101N-Δ*glnK_2_* | 4154 | - |
|  | Pop-out | HM26-K1K2 | 5187 | 5187 |

**Parental strain (HM26)**

3’

5’

*2132 bp*

**PCR 1**

**PCR 2**

*glnK1*

**PCR 3**

**PCR 4**

*glnK2*

**Pop-in A**

5’

*9061 pb*

3’

*glnK1*

*glnK2*

Δ*glnK2*

**Pop-out (HM26-K2, Δ*glnK2*)**

3’

5’

*glnK1*

*3165 pb*

Δ*glnK2*

**E**

**Parental strain (HM26)**

3’

5’

*2132 bp*

**PCR 1**

**PCR 2**

*glnK1*

**PCR 3**

**PCR 4**

*glnK2*

5’

3’

**Pop-in B**

*glnK2*

Δ*glnK1*

*glnK1*

*9058 pb*

**Pop-out (HM26-K1, Δ*glnK1*)**

3’

5’

*glnK2*

Δ*glnK1*

*4154 pb*

**D**


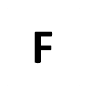
**Figure S3:** Southern blot analysis of *glnK* deletion strains. (**A**) DNA (3 μg) was *Asc*I digested, separated in a 1 % (w/v) agarose gel and probed against the 509-bp DIG-labeled PCR2 fragment amplified with primers glnK1-2F and glnK1-2R (Table 2). Lanes correspond to (1) DIG-labeled markers, (2) HM26 parental strain, (3) HM26 pMH101N-Δ*glnK1* pop-in, (4) HM26-K1, (5) HM26 pMH101N-Δ*glnK2* pop-in, (6) HM26-K2, (7) HM26-K1 pMH101N-Δ*glnK2* pop-in, (8) HM26-K1K2. (**B**) Southern blot confirmation of HM26-K1K2 strain in the same conditions as the blot in (A) but using as probe the 512-bp DIG-labeled PCR4 fragment amplified with primers glnK2-4F and glnK2-4R (Table 2). Lanes correspond to (1) DIG-labeled markers, (2) HM26-K1 parental strain, (3) HM26-K1K2. (**C**) Theoretical sizes of the *Asc*I digested DNA fragments that would hybridize with the probes in each genomic organization. -, not calculated. (**D**) Organization of the *glnK* genomic region during HM26-K1 knockout construction. Vertical arrows indicate *Asc*I restriction sites; purple double-ended arrows indicate *Asc*I digested DNA fragments that hybridize with PCR2 fragment. Genomic element sizes are not to scale, *amt* genes are not shown for simplicity. (**E**) Organization of the *glnK* genomic region during HM26-K2 knockout construction. Vertical arrows indicate *Asc*I restriction sites; purple double-ended arrows indicate *Asc*I digested DNA fragments that hybridize with PCR2 fragment. Genomic element sizes are not to scale, *amt* genes are not shown for simplicity. (**F**) Organization of the *glnK* genomic region during HM26-K1K2 knockout construction. Vertical arrows indicate *Asc*I restriction sites; purple double-ended arrows indicate *Asc*I digested DNA fragments that hybridize with PCR2 fragment, green double-ended arrows indicate fragments that hybridize with PCR4 fragment. Genomic element sizes are not to scale, *amt* genes are not shown for simplicity.

**Pop-in A**

5’

*11083 pb*

3’

*glnK2*

Δ*glnK2*

Δ*glnK1*

**Parental strain (HM26-K1, Δ*glnK1*)**

3’

5’

*glnK2*

Δ*glnK1*

*4154 pb*

**PCR 1**

**PCR 2**

**PCR 3**

**PCR 4**

*1384 pb*

**Pop-out (HM26-K1K2, Δ*glnK1* Δ*glnK2*)**

3’

5’

Δ*glnK1*

*5187 pb*

Δ*glnK2*


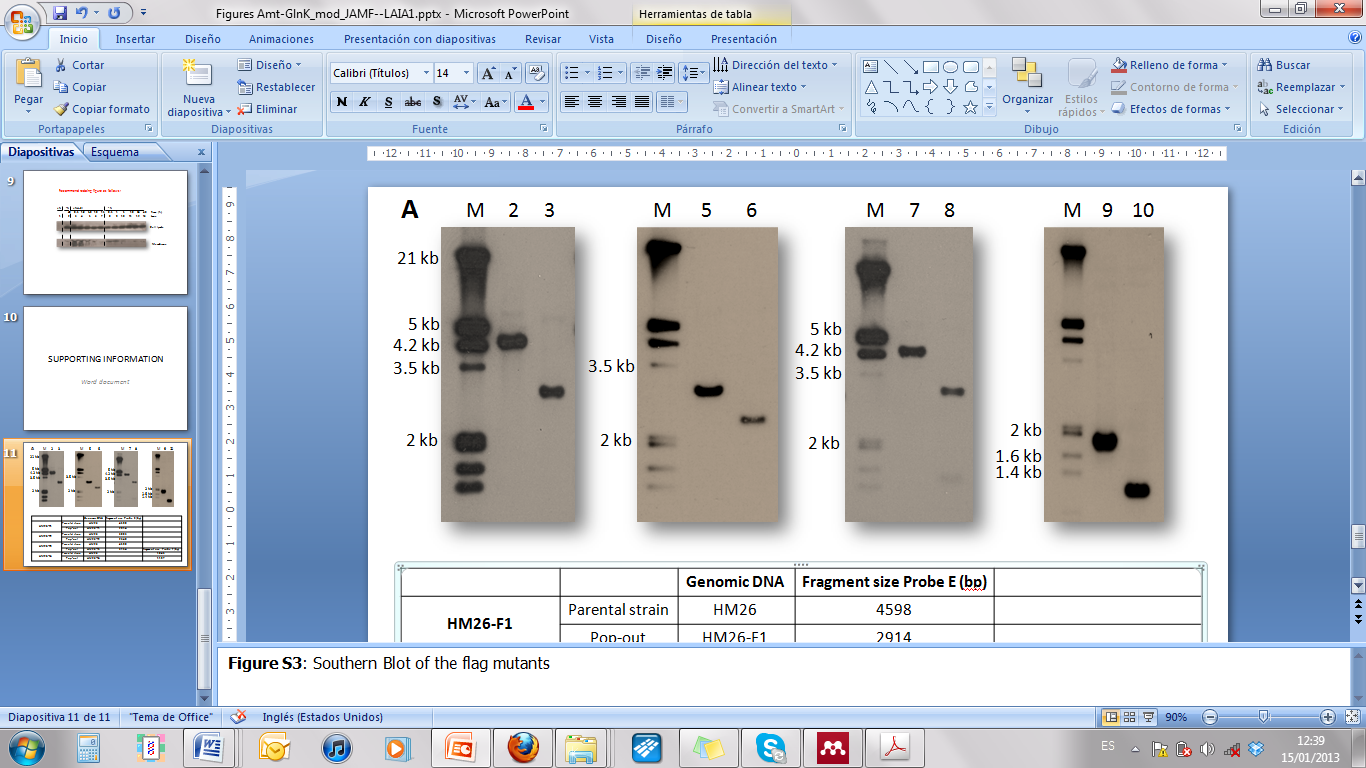


**B**

| **Mutant Strain** | **Parent vs. mutant** | **Genomic DNA** | **Fragment size Probe E (bp)** | **Fragment size Probe F (bp)** |
| --- | --- | --- | --- | --- |
| **HM26-F1** | Parent | HM26 | 4598 | - |
|  | Pop-out | HM26-F1 | 2914 | - |
| **HM26-F2** | Parent | HM26 | 3003 | - |
|  | Pop-out | HM26-F2 | 2340 | - |
| **HM26-F3** | Parent | HM26 | 4598 | - |
|  | Pop-out | HM26-F3 | 3154 | - |
| **HM26-F4** | Parent | HM26 | - | 1845 |
|  | Pop-out | HM26-F4 | - | 1197 |

***amt1*, N-terminal Flag-Tag**

**Parental strain (HM26)**

3’

5’

*amt2*

*4598 pb*

***Probe E***

*Kpn*I

*amt1*

*Sma*I

**Pop-out (HM26-F1)**

3’

5’

*amt2*

*2914pb*

***Probe E***

*Kpn*I

*amt1*

*Sma*I

***Flag-Tag***

*Kpn*I

***amt2*, N-terminal Flag-Tag**

**Parental strain (HM26)**

3’

5’

*amt2*

*3003 pb*

***Probe E***

*Kpn*I

*amt1*

*Mlu*I

**Pop-out (HM26-F2)**

3’

5’

*amt2*

*2340 pb*

***Probe E***

*Kpn*I

*amt1*

*Mlu*I

***Flag-Tag***

*Kpn*I


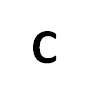


***amt1*, C-terminal Flag-Tag**

**Parental strain (HM26)**

3’

5’

*amt2*

*4598 pb*

***Probe E***

*Kpn*I

*Sma*I

*amt1*

***amt2*, C-terminal Flag-Tag**

**Parental strain (HM26)**

3’

5’

*amt2*

***Probe F***

*Kpn*I

*amt1*

**Pop-out (HM26-F4)**

3’

5’

*amt2*

*1197 pb*

*Kpn*I

*amt1*

***Probe F***

**Pop-out (HM26-F3)**

3’

5’

*amt2*

*3154 pb*

***Probe E***

*Kpn*I

*Sma*I

*amt1*

***Flag-Tag***

*Kpn*I

*Kpn*I

***Flag-Tag***

*Eco*RV

*1845 pb*

*Eco*RV


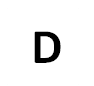


**Figure S4:** Southern Blot analysis of the *flag:amt* knockin strains. (**A**) DNA (3 μg) was digested and separated in a 1 % (w/v) agarose gel prior to hybridization. A *Kpn*I and *Sma*I digestion was used in the first and third blots, *Kpn*I and *Mlu*I were used in the second and *Kpn*I and *Eco*RV in the fourth. Probe E (224 bp, RT-Amt1For and RT-Amt1Rev primers) was used for the first, second and third blots; probe F (670 bp, Flaginv-8F and ProbeF-rev primers) was used for the fourth one (primers listed in Table 2). Lanes correspond to (M) DIG-labeled markers, (1) HM26 parental strain, (2) HM26-F1, (3) HM26, (4) HM26-F2, (5) HM26, (6) HM26-F3, (7) HM26, (8) HM26-F4. (**B**) Theoretical sizes of the digested DNA fragments that would hybridize with the corresponding probe in each genomic organization. -, not calculated. (**C**) Genome organization and digestion strategy for Southern blot check of the *flag:amt* mutants. The Flag-Tag is shown in blue, the 1 kb fragment cloned into the suicide plasmid in purple and the probe used plus the hybridizing fragments are shown as brown two-pointed arrows. DNA fragment sizes are not to scale and *glnK* genes are not shown for simplicity. (**D**) Genome organization and digestion strategy for Southern Blot check of the *amt:flag* mutants. The Flag-Tag is shown in light blue, the 1 kb fragment cloned into the suicide plasmid in pink and the probe used plus the hybridizing fragments are shown as two-pointed arrows, brown for Probe E and dark blue for Probe F. DNA fragment sizes are not to scale and *glnK* genes are not shown for simplicity.


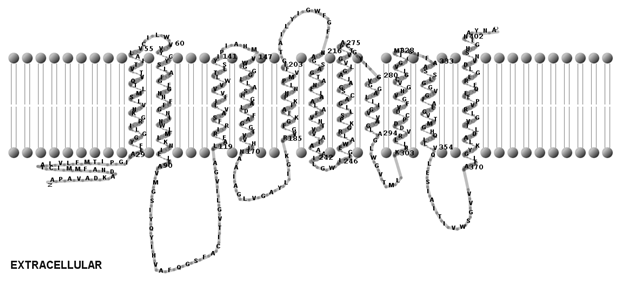

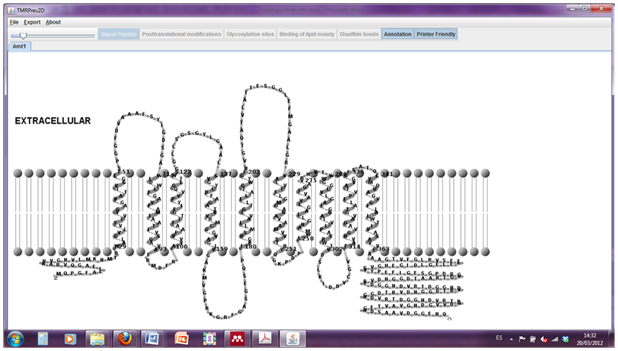


*N-terminus*

*C-terminus*

**A**


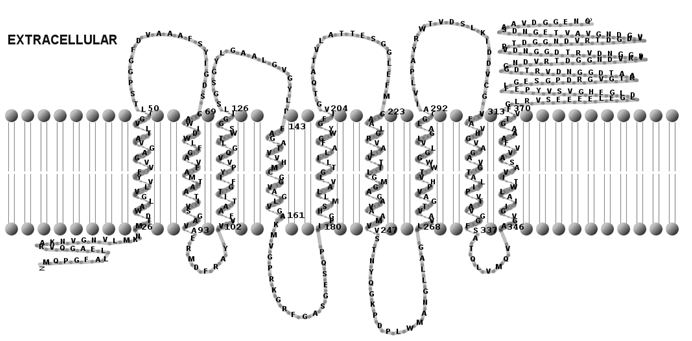


**B**

*N-terminus*

*C-terminus*

*N-terminus*

*C-terminus*


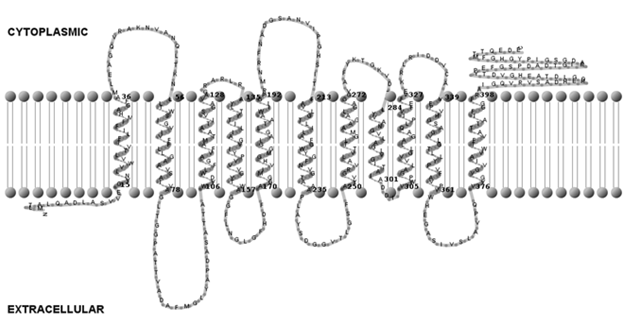


*N-terminus*

*C-terminus*

**C**

**D**

**Figure S5**: *H. mediterranei* Amt topology predictions. (**A**) Amt_1_ prediction with TMHMM, (**B**) Amt_1_ prediction with HMMTOP, (**C**) Amt_2_ prediction with TMHMM, (**D**) AmtB from *E. coli*, from resolved three-dimensional structure (PDB ID: 1U77).


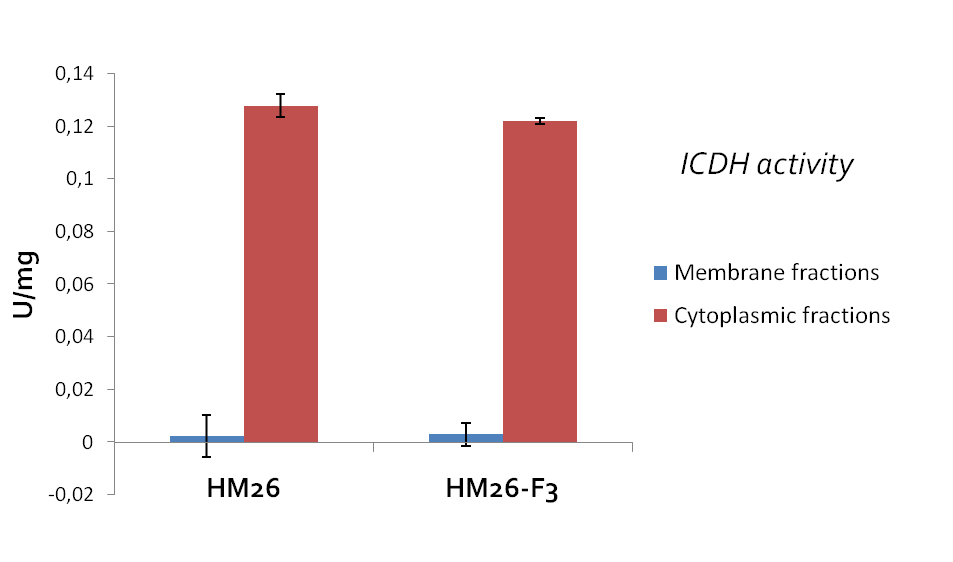


**Figure S6:** Membrane fraction purity control of IP samples. Membrane and cytoplasmic fractions from HM26 and HM26-F3 cells grown in complex medium (OD 1), nitrogen starved for 48 hours and finally submitted to an ammonium shock (150 mM, 1 hour), were tested for ICDH activity as described before for *Hfx. volcanii* (Camacho *et al.* 1995).


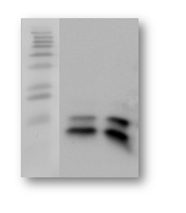


1 2 3

10 kDa

**15 kDa**

20 kDa

25 kDa

37 kDa

**Figure S7**: Anti-GlnK immunoblot of HM26 and HM26-F3 membrane fractions. HM26 and HM26-F3 were grown in complex medium (OD 1) and nitrogen starved for 48 h prior to exposure to 150 mM NH_4_Cl for 1 h. Cells were harvested and membrane fractions were separated by 15 % SDS-PAGE and anti-GlnK immunoblotted. Lanes correspond to (1) *Kaleidoscope Molecular Weight Marke*r*s* (*Bio-Rad*), (2) HM26-F3 resuspended membranes (10 μg), (3) HM26 resuspended membranes (10 μg). Exposure time: 15 min.


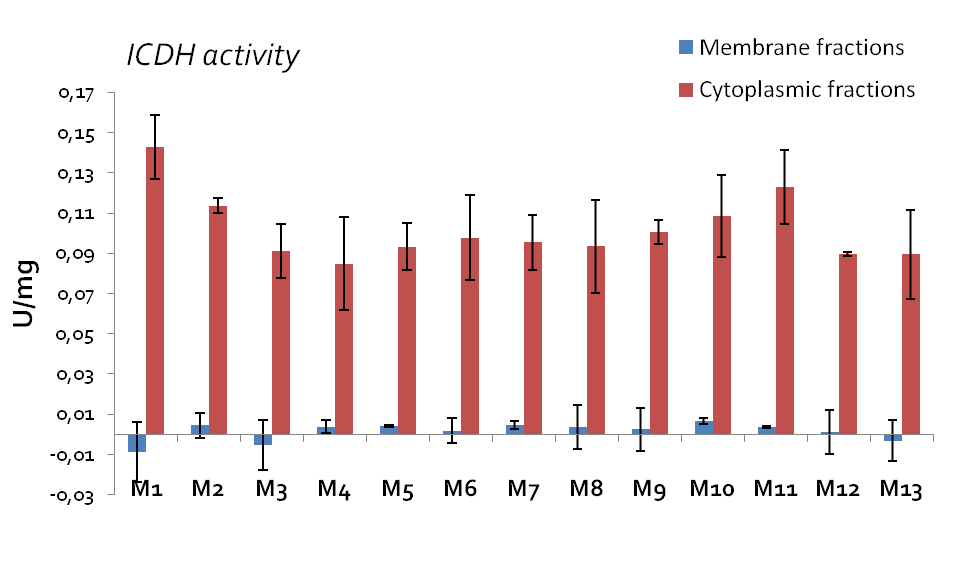


**Figure S8:** Membrane fraction purity control of the reversibility assay samples. Membrane fractions (see Figure 8) were tested for ICDH activity as described before for *Hfx. volcanii* (Camacho *et al.* 1995). Cytoplasmic fractions were also analysed as positive controls.

***amt1*** ------------------------------------------------------------------------------------------

***amt2*** ATGCTGACCGCACTTCAGGCAGACCTCGCCTCGGTCGTCGAGGGCGTGAATCTCGTGTGGGTCCTCACCGTCACGTTCCTCATCTTCTTC

***amt1*** ATGCAGCCGGGGTTCGCGCTCTTAGAGGCAGGGCAGGTACGCGCGAAAAACGTGGGGAACGTGCTGATGAAGAACATGACCGACTGGGCG

***amt2*** ATGCACGCCGGCTTCGCCATGCTCGAAGCCGGGCAGGTGCGTGCGAAGAACGTCGCAAATCAGCTCACGAAGAACCT-CCTGACGTGGAG

***** * ** ***** .* *.**.**.********.** *****.***** * .** :*** * *******.* .* *** **.*

***amt1*** -CTCGGCGTCCTCGTGTACTTCGTCGTCGGTGCGGGCGTGGCGACAATCGTCGGCGGCCTCACCTCGCCGGGCGGCTTCGACG-TTGCCG

***amt2*** TATCGGCGTCATCGTGTTCTTCCTCCTCGGTGCGGCCGTCTCGGCAATCGTCGCCGGACTTACCG-GCGGCCCGGCAACGACCGTCGCAG

.********.******:**** ** ********* *** **.********* ***.** *** ** * ****::**** * **.*

***amt1*** CCGCGTTCTCG------TAC---ATCGGCGACTCCGG------CGCGTGGATTGACTGGCTCTTCGGTGCCGTCTTCGCCATGACCGCCG

***amt2*** ACGCCTTCATGGGTCTCTACGCACCCGACGCCTCGGCGACGACCGCGTGGGTCGATTGGCTCTTCGGTGCCGTCTTCGCCATGACCGCCG

.*** ***: * *** . **.**.*** * *******.* ** **********************************

***amt1*** CCACCATCGTCTCCGGTGCCGTCGCAGAGCGCATGGATTTCCGTGCGTACGTCGTCTTCGCGGCGACCATCACGGGATTCATCTACCCCG

***amt2*** CCACCATCGTCTCCGGTGCAGTGGCGGGCCGCGCGAGGCTTCGTGCGTACCTGACCTACACCATCCTCATCGCGGGCGTCATCTACCCCG

*******************.** **.*. ***. *.. * ********* * . **:*.* . . ****.****. ************

***amt1*** TCGTACAGGGCCTGACGTGGTCCGGCGGCCTGCTCTCCGGAAGCGGCTATCTCGGTGCCGCGCTCGGCGTCGGCTACCTCGACTTCGCCG

***amt2*** TCGTCGTCGGCGTCACCTGGGCCGGTGGCTTCCTCAACGG---------TCTCG------------------GCTTCCACGACTTTGCCG

****. : *** * ** *** **** *** * ***:.*** ***** ***:**:****** ****

***amt1*** GTGCGACCGTCGTCCATATGTGCGGTGGCGTCGCCGGTCTCGTCGGCGCGAAGATGGTTGGCCCGCGCAAGGGTCGCTTCGGCGCGAGCG

***amt2*** GCGGCATGATTGTCCACGGAATGGGCGGCATCGCTGGTCTCACCGCCGCGTGGATTATCGGCCCGCGCATGAACCGCTTCAACGCCGACG

* * * .* ***** . .: ** ***.**** ******. ** ****:.*** .* **********:*.. ******..*** ..**

***amt1*** GTGAGAGCCAGC-CCATTCCGGGCCACTCGATGCTGCTCGCGGTCCTCGGGACGCTCATCCTCGCGTTCGGCTGGTACGGCTTCAACGTC

***amt2*** G-CAGCGCGAACGTCATCCCCGGCCACTCCATCACGTTCGCCGTCCTCGGGACGCTCATCCTCGCGTTCGGCTGGTACGGCTTCAACGTC

* **.** *.* *** ** ******** ** . * **** ************************************************

***amt1*** GGCACGCAGGCGACCGTCCTCGCAACCACCGAAAGCGGCG-GTCTGGAGTTCATGGGTGCCGCACTCGGCCGCGTCGCGCTCGTGACGAC

***amt2*** GGTACCGCCGCTGCACCACTCGCGTACAGTGACGGCGGCGTGACCCTCGG-CTCGTTCGCCTACGTCGGACGGGTCGCGCTCGTGACCAC

** ** . ** . *. .*****.:.** **..****** *:* .* *: * *** .. ****.** ************** **

***amt1*** CCTCGGCATGGGTGCCGGTGCGGTGGCCGCGATGGTCGTCTCCACGAACTACCAGGGTAAGCCCGACCCGCTCTGGATGGCGAACGGTCT

***amt2*** CCTCGGCATGGCCGCGGGCGCACTCGGCGCGGGCGGCGTCGCGTTCTACAAGACCGGCAAGGTCGACACGCTCTACGTCGCAAACGGCGT

*********** ** ** **. * * ****. * **** * : :**:* .. ** *** ****.******. .* **.***** *

***amt1*** GCTCGCCGGACTTGTGGCCGTCACGGGCGCTGTCCCGCACGTCACGTGGTGGGGTGGCCTCGTCCTCGGCGCACTCGGCGGCGCA-ATCG

***amt2*** TCTCGCCGGACTCGTCGGCATCACCGCCATCGCCGACGACATCGTCTGGCCCGGCGCTCTCGTCGTCGGGCTTCTCGCCGGCGCACAACT

*********** ** * *.**** * *. * * . **.**. *** ** * ****** **** :**** ******* *:*

***amt1*** TCCTGCCCGCGTACCGCTGGACCGTCGACTCGCTGAAAATTGATGACGTGTGCGGCGTCTTCGCCGTCCACGGCGTCGCCGGCGCGGTCG

***amt2*** TCCAATCGTCTTCGAGTTCGTCGAAAAGCGC-CTCCGCATCGACGACGTGTGTGCGGTCTTCCCCGTTCACGGCTCCGCGGGCGTTCTCG

***:. * * *. .* * *:* .:...* * ** ...** ** ******** * ****** **** ****** *** **** ***

***amt1*** GCACGGCCCTCATTCCGGTGTTCGCCGTCGGCG----GCTTTTCGGC-----------CACGCAACTGGTCATGCAGGTCGCCGGTGTCG

***amt2*** GGACGCTCCTGTACCCGGTGTTCGCCGTCCCCGTTTGGCACGAAGGCGCGTCCATCGTCTCGCTCGCCGTTCCGCAGGTGGTCGGCGTCG

* *** *** :: *************** ** **: :.*** *:***:. ** . ****** * *** ****

***amt1*** GCATCATCGCCCTGTGGACCATCGTCGCCTCGGCGGTCGTCTTCG-CGGCCGCTGGCACCGTCTTCGGCCTCCGCGTCTCGGAGGAAGAA

***amt2*** GCGTCATCGCCGTCTGGACCTTCGTCGCCACCACGGCAATCTTCGGCGGCTTCCG-CGCCATTGGACAGGTCCGTGTCTCCGCCGACCAC

**.******** * ******:********:* .*** ..****** **** * * *.**.* . . **** ***** *. **. *.

***amt1*** GAACTCGAAGGCCTCGATATCGGCGAACACGGCGTCTCGGTCTACCCAGAGTTCATCGGCGAATCGGGTCCCGACCGTGGCGTTGGAACG

***amt2*** GAGCGTCAGGGACTCGACACGGCCGAACACGGCGTCGACACCTACCCCGAATTCGGC------TCG---CCCGACG--------------

**.* *.**.***** * * ************* . . ******.**.***. * *** ******

***amt1*** CGCGCCGCAACTGACGGTGGGAACGATGTCCGGACTGACGGTGGGAACGATGTCCGGACTGACGGTGGGAACGATGTCCGGACTGACGGC

***amt2*** ----CCGACAC------------CGGTATCCGCGCCGACGG----------CTCCGGGATTCC-----------------------CTAC

***..** **.*.**** .* ***** *****..* .* * .*

***amt1*** GACGTCGTTGGCGACAACGGCGTGGCCGTGACTGAAGGCAACGATTCGGCCGCAGTCGACGGAGGTGAAAACCAATGA 1392

***amt2*** G-----------------------------------GGCATGGATTC-----ATGACGACACAG--GAGGACGAATAA 1371

* ****: ***** .:*:****. ** **..** ***.*

**Figure S9**: Alignment of Hfx. mediterranei amt genes showing the primers used in the RT-PCR analyses. The alignment was performed using ClustalW2, (*) indicates conserved nucleotides. The RT-PCR primers are highlighted: amt_1_ in pink (RT-Amt1For, RT-Amt1Rev), amt_2_ in blue (RT-Amt2For, RT-Amt2Rev).
